# Alfalfa and *M. truncatula* TILLING lines divergent for nutritive value-related traits enable the identification of novel candidate genes by genotyping by Sequencing approach

**DOI:** 10.1007/s12298-026-01750-2

**Published:** 2026-05-26

**Authors:** Maria Carelli, Nelson Nazzicari, Massimo Confalonieri, Carla Scotti

**Affiliations:** Council for Agricultural Research and Economics (CREA) - Research Centre for Animal Production and Aquaculture, Lodi, Italy

**Keywords:** Alfalfa, *Medicago truncatula*, Forage quality, Flowering time, Stem morphology, GBS analysis

## Abstract

Alfalfa (*Medicago sativa* L.) is the main protein producer among forage crops. Stem morphology and flowering time represent key traits controlling alfalfa leaf-to-stem ratio, and consequently its, forage nutritive value. Breeding programs for varying flowering timing and stem internode length, including selfing, produced six alfalfa narrow-based ‘lines’ showing traits like early (EF) and late (LF) flowering, and alfalfa synthetics with divergent stem morphology (long/short internode length, LI vs. SI). Two *M. truncatula* (*Mt*) TILLING mutant lines segregating for flowering time were also included. These materials were used to identify the genetic variation (SNPs) underlying these traits using Genotyping by Sequencing (GBS). Fifty-seven SNPs were found differentiating alfalfa EF and LF lines, 13 of which were common to at least two couples and five laying on exons and producing amino acid (a.a.) changes. Forty-nine and sixteen SNPs were detected, differentiating EF and LF individuals in the *Mt* lines, twenty and seven of which laying on exons and causing amino acid changes. The analysis of LI and SI synthetics identified 14 SNPs one of which bringing to amino acid change. Transcription factors and genes involved in DNA, RNA and protein modification, regulation and remodelling, sugar and lipid biosynthetic pathways were the most represented classes. The functional relationship between nucleus and organelles, in particular chloroplasts, emerged as a key point in defining the different alfalfa earliness profile. Some of the identified genes show a possible correlation with the traits studied; the pentatricopeptide repeat-containing proteins, MtrunA17Chr1g0199691 (flowering time) and MtrunA17Chr3g0128551 (stem morphology) emerge as promising candidates for the *Medicago* spp. genetic improvement.

**Supplementary Information:**

The online version contains supplementary material available at 10.1007/s12298-026-01750-2.

## Introduction

Alfalfa (*Medicago sativa* L.) is the main protein producer among forage crops yielding 2.112 t ha^−1^ of protein considering 13.2 t average dry matter yield (DMY) with average protein content of 16%. However, unlike grain legumes, in alfalfa the entire plant (leaves and stems) contributes to protein production and plants are cultivated in the dense sward condition (meadow). Leaves concentrate the largest part of alfalfa protein production. The leaf layer of the upper stem nodes receiving the highest light radiation showed a rather stable protein content (around 33–36%); leaf protein content is reduced (27–30% in green leaves) in the more basal stem nodes because of leaf senescence processes related to light radiation decrease in dense sward conditions, and internode maturity (Rotili et al. 1992). The protein content of alfalfa stems showed a variation during the productive cycle from around 20–22% in the early regrowth stage to a progressive decrease at harvesting time around 13–15% (Rotili et al. 1992). Besides, the vegetative-to-reproductive transition in alfalfa involves changes in a wide range of developmental traits such as stem elongation, apical dominance, lateral branching, resource allocation, and maturity (Adhikari et al. 2019). In particular, alfalfa harvesting at different maturity stage – e.g., from early bud to full bloom stage – had a large impact on protein and carbohydrate fractions, in vitro rumen degradability and energy value (Yu et al. 2003; Palmonari et al. 2014).

The investigation on flowering time control in *M. truncatula* has been mainly driven by the deeper knowledge of *Arabidopsis thaliana* (Jaudal et al. 2020; Fudge et al. 2018; Cheng et al. 2020). Negative regulators of photoperiodic flowering in *Medicago* have been identified: *MtCDFd1_1* (MtrunA17_Chr6g0460731), part of the CYCLING DOF FACTOR *MtCDF* genes, *via* the repression of *MtFTa1*, the sole target of the vernalization pathway and rapidly upregulated in leaves in response to LD photoperiod (Zhang et al. 2019), and the *MtTFL1* encoded protein (Guo et al. 2020a). Besides, the alfalfa photoreceptor blue-light and circadian clock-responsive *MsFKF1* (MtrunA17_Chr8g0391901) was found to delay the flowering process by regulating the *MsFTa1* pathway *via* the transcription inhibition of *MsE1* (Jiang et al. 2024). The serine/threonine protein kinase genes *MsK-1*,* MsK-2,* and *MsK-3* have been also proposed to take part in the phytochrome light signal transduction *via* transcription factor (TF) phosphorylation in *M. sativa* (Pay et al. 1993).

Most of the identified *Medicago* flowering genes display TF activity with the function of integrating and channeling environmental signals influencing flowering transition. However, plant endogenous signals - such as carbohydrate status, gibberellin metabolism, developmental stage and the autonomous floral promotion pathway (Peer et al. 2021) - and different regulation levels - including epigenetic, transcriptional, posttranscriptional, and posttranslational (Quiroz et al. 2021) - also interact in flowering control, increasing its complexity. Conversely, some of these genes were found to influence also stem architecture by promoting primary axis elongation (*MtSOC1a*, Jaudal et al. 2018; *MtPHYA*, Jaudal et al. 2020), stem internode elongation (*MtSOC1b* and *MtSOC1c*, Fudge et al. 2018), plant height and gibberellin levels (*MsFKF1*, Jiang et al. 2024) or by repressing primary stem elongation (*MtCDFd1_1*, Zhang et al. 2019). In fact, light is an important environmental factor influencing shoot architecture often *via* the interaction with phytohormone signaling pathway (Wang et al. 2023; Li et al. 2021).

Breeding programs in *Medicago sativa* (*Ms*) were then implemented at Lodi Institute for the traits stem morphology and timing of flowering that proved to be effective at obtaining alfalfa lines with divergent earliness (early/late flowering) and divergent stem morphology (long/short internode length) (Scotti et al. 2007; Depedro et al. 2016; Pecetti et al. 2017). In our hypothesis, such genetic materials would represent an effective tool to investigate the genetic variation (Single Nucleotide Polymorphisms, SNPs) at the basis of the traits selected by means of Genotyping-by-Sequencing (GBS) approach (Elshire et al. 2011).

The diploid self-fertile annual *Medicago truncatula* (*Mt*) has emerged as one of the model species for legumes (Pecrix et al. 2018), promoting the assembly of GBS data in closely related taxa, especially alfalfa. In a mutant *Mt* TILLING collection established at Lodi Institute (Porceddu et al. 2008), M3 segregating lines were used for phenotypic evaluation and seed increase. The hypothesis was that full-sib M3 individuals segregating for specific traits of interest within each line would maximize the probability to identifying the SNPs underlying the phenotypic differences observed (Carelli et al. 2013).

In this paper, we investigate how alfalfa ‘lines’ with a great genetic homogeneity (narrow genetic base with reduced heterozygosity) genotypically and phenotypically divergent for complex traits - time and dynamics of flowering and stem morphology - could be effective for associating the observed SNPs to the target traits, ultimately providing new information exploitable for alfalfa improvement. Two *M. truncatula* mutant lines segregating for time of flowering are also investigated to provide new insight into the relationship of flowering time determinants in *Medicago* closely related species differing in plant biology.

## Materials and methods

### *Medicago sativa*

#### Alfalfa ‘lines’ with divergent earliness (early/late flowering)

Plant material is described in Depedro et al. (2016). Briefly, a breeding method based on two generations of selfing (S_2_) and selection (Rotili et al. 1999) was used to obtain S_2_ partly-inbred families with high DMY and divergent earliness estimated by earliness index (early flowering plants, EF, with earliness index> mean+1 standard deviation (*s*); late flowering, LF, with earliness index <mean-1s, at family level). By crossing four of these families according to a diallelic scheme, we obtained two subgroups of six S_2_ x S_2_ Simple Hybrids (SHs). We advanced the SHs for two generations (syn2 and syn3) applying a conservative selection at each generation to have 2-constituent S_2_ synthetics (2S_2_syn3) genetically in equilibrium (‘lines’), consistently expressing the EF and LF profile (Online Resource 1). The corresponding 4-constituent synthetics EFsyn and LFsyn were also developed by among-SH manual crossing (4S_2_syn2) and advanced to 4S_2_syn3 (Online Resource 1).

**Fig. 1:**
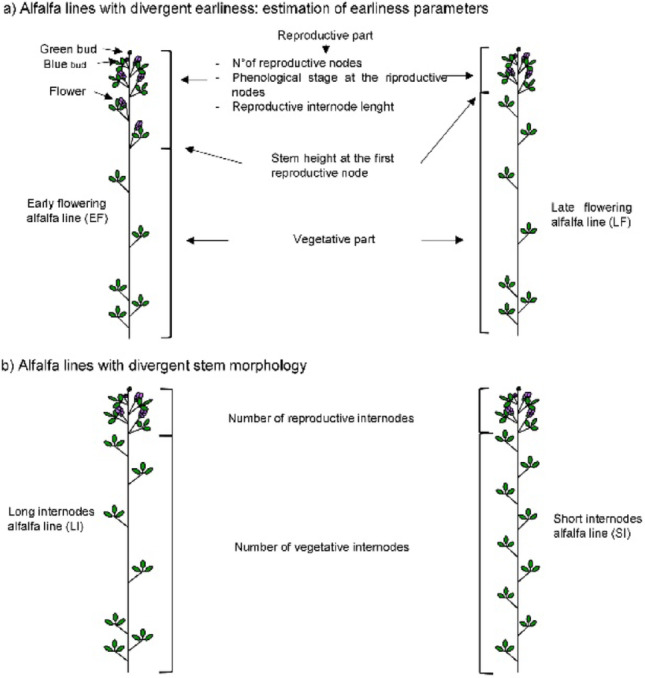
Breeding of alfalfa for divergent earliness and stem morphology

Sixty plants per 2S_2_syn3 ‘line’ and 120 plants for synthetics arranged in a randomized block design with 4 blocks were grown individually in tube-plots 80 cm high x 5 cm diameter under a rain-out shelter in a 1-year trial at Lodi Centre, Italy. Parameters were recorded on single plant basis along harvests 1–5 (DMY) and 2–5 (earliness). Earliness was estimated on the three highest stems per plant by the parameters: earliness index, i.e. sum of the reproductive nodes each weighted by the phenological stage, from flower to green bud; total stem height; stem height at the first reproductive node (Fig. 1). The EF1- EF6 and LF1- LF6 ‘lines’ and the corresponding EF and LF 4S_2_syn3 synthetics used for GBS were represented each by bulks of 4–9 and of 10–15 chosen plants respectively (Online Resource 1).

#### Alfalfa synthetics with divergent stem morphology (long/short internode length)

Two generations of selfing (S_2_) and selection for high DMY and stem height/diameter and divergent average internode length of the main stem (short internode plants, SI, with internode length < mean-1*s*; long internode plants, LI, with internode length > mean+1*s*, Fig. 1) were applied to the progenies of plants from somatic hybridization *M. sativa* x *M. falcata* (Téoulé 1983) backcrossed to *M. sativa.* The resulting S_2_ selected plants were manually polycrossed to obtain partly-inbred syn1 and syn2 generation synthetics (SIsyn and LIsyn) (Scotti et al. 2007) and advanced to syn4 by insect pollination. Three pools of 150 two-month-old plantlets each from the syn4 generation of SIsyn and LIsyn were used for GBS analysis. The same seeds were used for a 2-year comparison in tube-plots 80 cm high x 20 cm diameter (20 plants/plot, density equivalent to 600 plants m^−2^) organised in a randomized block design with four blocks at Lodi Centre. Stem morphology was estimated in harvests 2 to 4 by the parameters: stem total height, stem height at the first reproductive node, number of vegetative and reproductive internodes, average vegetative and reproductive internode length, recorded on the main stem per plant. SIsyn4 and LIsyn4 were represented by eight plots each.

#### Medicago truncatula TILLING lines

Twenty mutagenized 3_rd_ generation (M3) seedlings per line were transplanted in tube-plots 80 cm high x 5 cm diameter (two plants/tube) under a rain-out shelter; days from transplanting to the appearance of the first open flower (DAT) were recorded at a single plant basis together with a visual estimation of plant vigour and phenotypic observations. *M. truncatula* lines having plants with medium-high and consistent vigour and delay of flowering time were recorded as putative mutants.

#### DNA isolation

DNA was extracted from leaves of single plants or bulks of plants using the GenElute plant genomic Miniprep kit (Sigma-Aldrich). In the case of alfalfa ‘lines’, an identical amount of leaf tissue was collected from the plants representing each ‘line’ and bulked for DNA extraction. In the *M. truncatula* segregating line 2345, the leaves of the three LF mutant plants were bulked while wild type plants were extracted individually; in the *M. truncatula* homozygous line 1739, all the plants were extracted individually and the DNA of cv. Jemalong genotype 2HA10-9 was used as a control.

#### GBS library construction and sequencing

DNA samples were sent to The Elshire Group Ltd. laboratory (Palmerston North, New Zealand) for sequencing. GBS data were generated according to Elshire et al.’s method (Elshire et al. 2011) and sequenced on Illumina X Ten platform with 150 bp paired-end reads.

#### Sequence analysis and identification of candidate genes

SNP calling was executed using the Legpipe2 pipeline (Nazzicari et al. 2024).

#### *Medicago sativa*

The full resequencing obtained by Long et al. 2022 was used as reference genome. For alignment, the longest copy of each chromosome was selected. The variants were quality-filtered (phred score > = 40) and minimum total reads > = 20).

The samples were genotyped as bulked genomic material coming from a collection of different individuals. As such, it is impossible to perform actual allele dosage estimation, we encoded the SNP information via a matrix of allele frequencies, as:

$$ AF_{{ij}} = \frac{{alt_{{ij}} }}{{(ref_{{ij}} + alt_{{ij}} )}} $$

where the allele frequency AF for SNP i and sample j is the number of observed alternative alleles over the total observed alleles. AF is a number between zero (all alleles are as reference genome) and one (all alleles are alternative to the reference genome).

All data points above 0.1 and below 0.9 were removed. Only markers with zero (for flowering date) or one (for internode length) missing data points were considered.

For divergent earliness, we compare each couple of divergent lines (EF1-LF1, EF2-LF2, etc.) with the corresponding couple of synthetics (EFsyn-LFsyn); only the SNP markers showing a consistent pattern, i.e. same allele on one phenotypic group and the opposite allele on the other group, were selected. This resulted in six lists of markers present both in a single line and in the corresponding synthetics.

For internode length, we selected the markers having the same allele on all short-internode samples and the opposite allele on all long-internode samples.

For only the selected marker, we extracted the 50-bp flanking sequence on either side from the *M. sativa* reference genome and mapped the resulting 101-bp fragment to the annotated *M. truncatula* genome (https://medicago.toulouse.inra.fr/MtrunA17r5.0-ANR/). If the 101 bp sequence aligned with more than one gene all alignments are reported.

#### *Medicago truncatula*

The annotated *M. truncatula* reference genome (Pecrix et al. 2018) was used. The obtained variants were filtered for quality (phred score > = 40) and minimum total reads per SNP (6). The final SNP calling was done using *updog* software (Gerard et al. 2020). SNPs were filtered for estimated proportion of misclassification < 5% and estimated allele bias in [e^−1^, e]. Finally, for each set (either 1739 or 2345 line), markers were allowed to have a single missing data point.

For the two sets, we extracted the markers showing statistically significant differences between the reference line (control) and the remaining part of set (mutant). We computed a statistical score as follows:

$$ DC_{i} = \sum_j \left| {alt_{ij}}-alt_{REF} \right| $$

where the difference count (DC) for SNP is computed by summing the absolute value of the difference in alternative allele count between the reference line and each sample j in the set. Remembering that the alternative allele count is a number between zero and two, and that the reference line is homozygous on all considered markers, DCs are thus defined between zero (all samples are homozygous for the marker and carry the same allele of the reference sample) and twice the number of samples in the set (all samples are homozygous for the marker and carry the alternative allele). For both sets, the empirical cumulative distribution of the DC scores was estimated via a permutation test and an empirical *p*-value corresponding to each possible value of DC was derived. Only markers with an estimated* p*-value < 0.05 were retained.

#### Gene ontology (GO) analysis

The 91 genes identified carrying SNPs in EF vs. LF *M. sativa* lines or *M. truncatula* individual plants were functionally classified according to Blast2GO (https://www.ebi.ac.uk/QuickGO).

The gene ontology (GO) enrichment analysis was performed using the tool ShinyGO v0.81 (http://bioinformatics.sdstate.edu/go/), (Ge et al. 2020) with a 0.06 FDR cutoff.

#### Statistical analyses

Analyses of variance (ANOVA) on plant DMY and phenotypic parameters were performed using the General Linear Model (GLM) procedures of SAS software version 8e (SAS Institute Inc.); linear contrasts were used for comparison of specific means.

#### Protein structural disruption analysis

We evaluated in silico the potential of the observed SNPs to induce structural disruption in the produced proteins.

For each considered SNP, a protein primary structure was extracted, corresponding to the wild-type version. The chain of amino acids was processed using Alphafold server v3 (Abramson et al. 2024) to obtain the tertiary, 3D-structure of the protein. The most probable model (“*model_0*”) was extracted and fed to DUET server (Pires et al. 2014a) together with information describing the amino acid substitution. For each SNP, three metrics describing the impact of the mutation on the protein structural stability were obtained. All metrics are referred to variations of ΔΔG, whereas ΔG is the Gibbs free energy of the folding, expressed in kcal/mol. Given the definition:

$$ \Delta \Delta G = \Delta G_{{wild - type}} - \Delta G_{{mutant}} $$

Negative values of ΔΔG imply that the amino acid change makes the protein more unstable. All metrics considered, mCSM (Cutoff Scanning Matrix), SDM (Site-Directed Mutator) and DUET reflect different underlying models of ΔΔG estimation (Pires et al. 2014a, b; Worth et al. 2011). For all metrics, we considered values below − 0.5 kcal/mol as suggestive, and below − 1.0 kcal/mol as strongly suggestive for a potential structural disruption. For *M. truncatula* the wild-type protein was the one found in Jemalong genotype 2HA10-9 for 1739 line and in wild-type plants for 2345 line. For *M. sativa* we considered as wild type the proteins corresponding to either early flowering (EF) or long internodes (LI) traits.

## Results

### Phenotypic characterization of *Ms* and *Mt* lines

#### Medicago sativa

##### Alfalfa ‘lines’ with divergent earliness (early/late flowering)

The six LF ‘lines’ and the LFSyn showed, on average, significantly lower values for the three earliness parameters, a similar stem height and a significantly higher DMY per plant compared to EF ‘lines’ and EFSyn. This indicated the effectiveness of the divergent selection for earliness applied in the selfing phase and maintained in the successive crossing phase (Table 1, Online Resource 2).

**Table 1:** *M. sativa* synthetics with (A) divergent earliness (early/late flowering): DMY and parameters estimating earliness at single plant basis (means of harvests 1–5 for DMY and 2–5 for earliness traits); (B) divergent stem morphology (short /long internode length): DMY and parameters estimating stem morphology at single plant basis (means of harvests 2–4)

A) Ms synthetic syn3 generation (mean)	DMY (g)	Stem height (cm)			Stem reproductive part (%)	No. of reproductive nodes	Earliness index
Early flowering	1.69 b	51.72 a			4.35 a	2.47 a	12.94 a
Late flowering	1.95 a	51.62 a			1.29 b	1.64 b	4.70 b

B) *Ms* synthetic syn4 generation			No. of vegetative internodes	Vegetative internode length (average)			
Short internode	2.47 *a*	58.83 *b*	10.45 *a*	4.95 *b*	4.95 *b*	3.67 *b*	5.38 *b*
Long internode	2.51 *a*	65.63 *a*	9.22 *b*	6.05 *a*	9.27 *a*	4.88 *a*	8.83 *a*

Mean separation by SNK test: means with different letters in the same column are different at *P* < 0.05 or *P* < 0.01 (italic)

Considering the selective effect within each cross, no significant difference in earliness parameters was found in 1 × 2 cross, which originated from two non-dormant parents, but a reduction in stem height and DMY in EF1 compared to LF1 (Online Resources 2, 3). In non-dormant vs. dormant 2 × 7 cross, only the number of reproductive nodes differed in EF5 compared to LF5 and a significant decrease in DMY and stem height was present in EF5. On the contrary, non-dormant vs. dormant 1 × 5, 2 × 5 crosses and EFSyn and LFSyn synthetics displayed highly significant differences for all the earliness parameters with similar stem height and/or DMY in early and late flowering subgroups (Online Resources 2, 3).

##### Alfalfa synthetics with divergent stem morphology (long/short internode length)

The two SIsyn4 and LIsyn synthetics clearly differed for stem morphology parameters, recorded at single plant basis in density condition along harvests 2 to 4 of the sowing year, in spite of a similar DMY (Table 1). In fact, the number of vegetative internodes in SIsyn4 was one unit higher than in LIsyn4, resulting in a significant decrease in the average internode length in SIsyn4. Total stem height in SIsyn4 was significantly reduced compared to LIsyn4 suggesting a decrease in internode elongation rate in SIsyn4 (Table 1). Then, a prolongation of the stem vegetative node production and a slight delay in stem switching to the reproductive phase, also evidenced by lower values in all the earliness parameters pertaining to reproductive nodes (Online Resource 4), characterized the SIsyn4 with respect to LIsyn4.

### Medicago truncatula TILLING lines

**Fig. 2:**
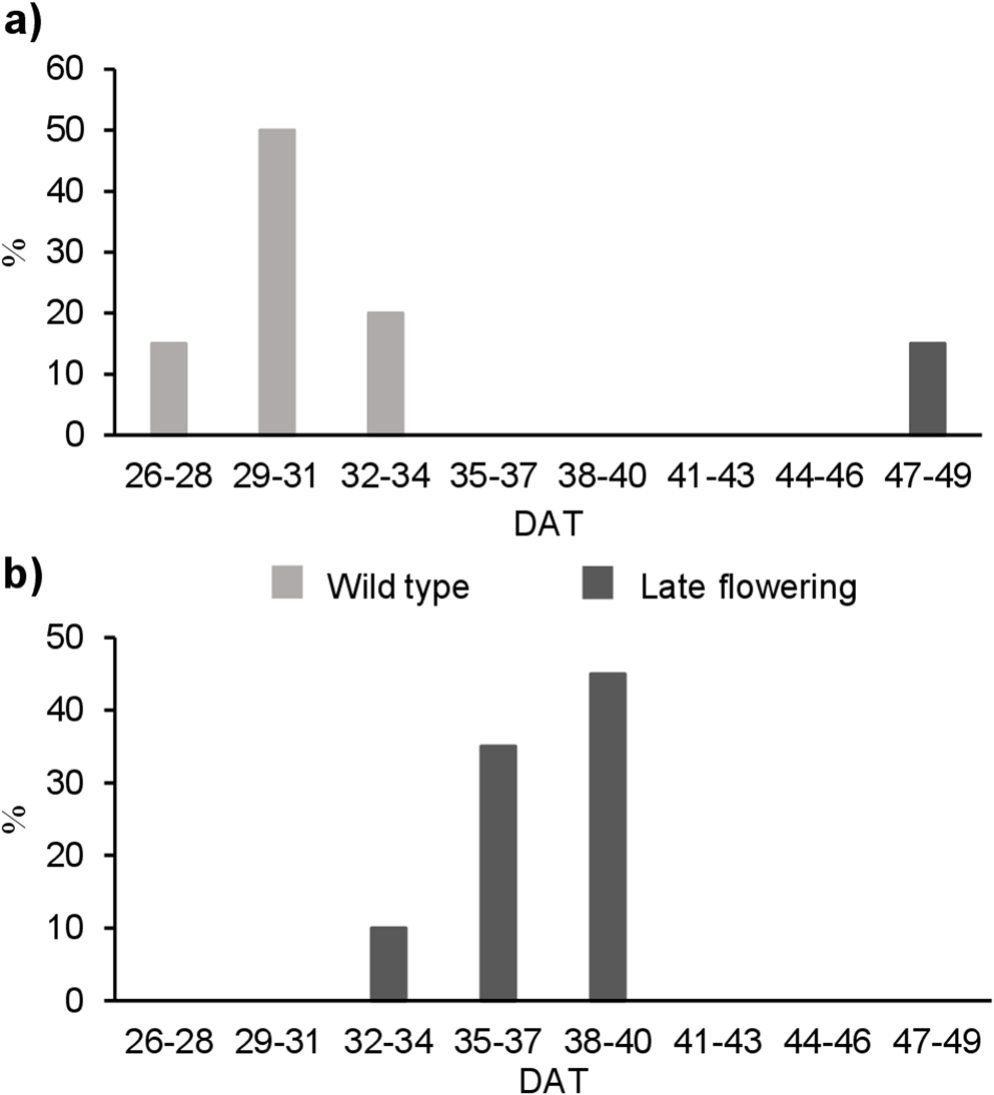
Percentage distribution of flowering date expressed as day after transplanting (DAT) in the plants of *M. truncatula* lines:** a** segregating line 2345;** b** homozygous line 1739

Two lines delayed in flowering time were identified. In the segregating line 2345, three out of twenty plants were significantly delayed in flowering (average DAT 47.67 ± 0.33) compared to the other 17 plants (average DAT 30.00 ± 0.51) and thus considered putative mutants for the trait. In the homozygous line 1739, eighteen plants with medium-high vigour showed a significant delay in flowering (average DAT 37.11 ± 0.44) with respect to the 17 wild type (wt) individuals of line 2345 (Fig. 2). The remaining two plants of 1739 line showed a severe flowering delay (50 and 63 DAT, respectively) but a retarded-in-growth phenotype and were then discarded from further analysis.

### Sequence analysis and identification of candidate genes

The described SNP calling procedure resulted in an allele frequency matrix of 84,095 markers, further filtered down to 2524 and 3587 for flowering date and internode length, respectively.

The identified SNPs were located in genes introns or exons, some of which resulting in amino acid (a.a.) changes, in UTR regions or in intergenic regions. SNPs leading to a.a. changes can directly impact protein function. However, also SNPs in introns could lead to changes in splicing, while SNPs in intergenic regions might affect transcription regulation. Therefore, all the identified SNPs can be taken into consideration for breeding purposes.

### Medicago sativa

#### Alfalfa ‘lines’ with divergent earliness (early/late flowering)

Seventy-seven SNPs differentiating LF from EF were identified in the six comparisons, some of these being common to two or more lines (Online Resource 5).

The 14 SNPs present in at least two comparisons are shown in Table 2. For the two SNPs no alignment was found in *M. truncatula* A17 r5.0 genome; three SNPs were located in intergenic regions and seven in exons, five of which resulting in amino acid changes in the corresponding protein sequences. Two among these non-synonymous SNPs (nsSNPs) lay in genes putatively related with male (pollen) fertility, MtrunA17_Chr2g0292681 coding for the protein POLLENLESS 3 and MtrunA17_Chr3g012031 coding for the long chain acyl-CoA synthetase 8. MtrunA17Chr3g0084091 is a plasma membrane-localized transporter and the two cytosolic genes MtrunA17Chr4g0075951 and MtrunA17Chr4g0076431, coding for a putative WEB family protein, showed 44% and 46% identity, respectively with At1g12150 related to *AtPMI2* (At1g66840) involved in blue light-induced chloroplast movement (Kodama et al. 2010).

**Table 2:** Alfalfa SNPs for flowering time

Chromosome	Position	Gene (*M. truncatula* A17 r5.0 genome)	SNP position	Gene function (*M. truncatula* A17 r5.0 genome)	AA change EF/LF	Line pairs with SNP
chr1_1	29,345,093	MtrunA17Chr1g0191161	4° exon	Putative WIYLD domain-containing protein		EF1-LF1; EF2-LF2
chr1_1	35,205,946	MtrunA17Chr1g0171471	3° exon	Putative glycerol-3-phosphate dehydrogenase		EF4-LF4; EF5-LF5; EF7-LF7
chr1_1	69,857,191		Intergenic region			EF2-LF2; EF3-LF3; EF4-LF4; EF6-LF6
chr2_1	17,246,013	MtrunA17Chr2g0292681	4° exon	Putative tetratricopeptide repeat protein POLLENLESS 3	Thr/Ala	EF2-LF2; EF4-LF4; EF6-LF6
chr2_1	68,555,885	MtrunA17Chr2g0323941	11° exon	Putative non-specific protein-tyrosine kinase RLK-Pelle-LRR-V family		EF2-LF2; EF5-LF5
chr3_1	91,883,134	MtrunA17Chr3g0120731	4° exon	Putative long-chain-fatty-acid-CoA ligase	Stop/Glu	EF1-LF1; EF3-LF3; EF4-LF4; EF6-LF6
chr4_1	74,648,725		Intergenic region			EF1-LF1; EF6-LF6
chr4_1	76,319,199	MtrunA17Chr3g0084091	5° exon	Putative EamA domain-containing protein	Leu/Ser	EF1-LF1; EF2-LF2; EF5-LF5
chr6_1	81,846,377	No alignment found				EF3-LF3, EF6-LF6
chr7_1	32,237,060 32,237,110	MtrunA17Chr7g0240901	2° exon 2° exon	Putative transcription factor interactor and regulator CCHC(Zn) family	Gln/Glu No change	EF1-LF1; EF6-LF6
chr7_2	75,842,177	No alignment found				EF2-LF2; EF5-LF5
chr8_1	50,421,996	MtrunA17Chr8g0368371	Intergenic region			EF1-LF1; EF4-LF4; EF5-LF5
chr8_1	89,476,522	MtrunA17Chr4g0075951 MtrunA17Chr4g0076431	1° exon 2° exon	Putative WEB family protein Putative WEB family protein	Thr/Ala Thr/Ala	EF1-LF1; EF3-LF3

Comparisons between early (EF) and late (LF) flowering lines and the corresponding synthetic line (EFsyn and LFsyn); only SNP present in at least two comparisons are reported. AA change: amino acid change found in protein sequences

The phytochrome *MtPHYB* (MtrunA17Chr2g0294361), a photoswitch in light signaling pathways among which flowering time, was interested by two SNPs in EF5-LF5 comparison but not resulting in amino acid changes (Online Resource 5). Similarly, the SNP in MtrunA17Chr7g0240901, a putative TF interactor and regulator CCHC(Zn), did not produce an amino acid change (Table 2).

#### Alfalfa synthetics with divergent stem morphology (long/short internode length)

Fourteen SNPs significantly differentiating SI and LI synthetics were identified in alfalfa synthetics (Table 3, Online Resource 6). For one SNP no alignment was found in *M. truncatula* A17 r5.0 genome; three SNPs were located in introns, four in the UTR regions, one in the intergenic regions and five in exons, one of which resulted in amino acid change in the protein kinase RLK-Pelle_LRR-III (Table 3). The most represented gene class (four genes out of eleven) was protein regulation and in particular protein kinases (Table 3).

**Table 3:** Alfalfa SNPs for internode length

Chromosome	Position	Gene (*M. truncatula* A17 r5.0 genome)	SNP position	Gene function	AA change LI/SI
Chr1_1	49,970,659	MtrunA17Chr1g0181651	1° exon	Putative non-specific serine/threonine protein kinase	
Chr2_1	12,742,137	MtrunA17Chr2g0288441	1° intron	Putative ATP synthase, F1 complex, gamma subunit	
Chr2_1	66,246,619	MtrunA17Chr2g0321101	1° exon	Putative protein kinase AGC-RSK-2 family	
Chr2_1	69,548,749	No allignement found			
Chr3_1	64,548,426	MtrunA17Chr3g0111011	3’UTR region	Putative glutathione dehydrogenase	
Chr3_1	71,102,283		intergenic region		
Chr4_1	7,283,617	MtrunA17Chr4g0036011	1° exon	Putative arginine decarboxylase	
Chr4_1	46,503,437	MtrunA17Chr4g0035881	1° exon	Putative tripeptidyl-peptidase II.	
Chr5_2	6,391,112	MtrunA17Chr3g0128551 (Medtr3g093710)	2° exon	Putative protein kinase RLK-Pelle_LRR-III family	Glu/Lys
Chr5_2	10,624,251	MtrunA17Chr5g0401551	3’UTR region	Putative tetratricopeptide repeat protein SRFR1	
Chr6_1	97,856,076	MtrunA17Chr1g0148461	3’UTR region	Putative protein kinase RLK-Pelle-WAK family	
Chr7_2	57,922,206	MtrunA17Chr7g0244271	5’UTR region	Putative NSF attachment protein	
Chr7_2	76,403,993 76,404,036	MtrunA17Chr7g0262051	5° intron 5° intron	Putative Ras GTAase-activating protein-binding protein	

Comparisons between short (SI) and long (LI) internode lines. AA change: amino acid change found in protein sequence

### Medicago truncatula TILLING lines

A total of 139 and 96 SNPs differentiating EF vs. LF plants were identified in line 1739 and 2345, respectively (Online Resources 7, 8). Only SNPs differentiating putative mutants from control plants with a difference of at least 90% and a significant DC score (*p*-value < 0.05) were retained.

In 1739 homozygous line, among the 49 SNPs selected, 13 SNPs were located in introns, two in the 3’ UTR region, two in repeat regions, three in intergenic regions and 29 in exons. Twenty-two SNPs located in the exons resulted in amino acid changes in the corresponding protein sequences (Table 4).

**Table 4:** *M. truncatula* 1739 line selected SNPs

Chromosome	Position	Gene (*M. truncatula* A17 r5.0 genome)	SNP position	Gene function	AA change Contr/LF
1	7,190,715	MtrunA17Chr1g0155351	2° intron	Pentatricopeptide repeat-containing protein	
1	9,763,490	MtrunA17Chr1g0158991	2° exon	Phosphate transporter 4 (AY116211)	Val/Ala
1	40,315,896	MtrunA17Chr1g0191211	1° exon	Putative protein	
1	46,672,493	MtrunA17Chr1g0200031	3° exon	Putative protein	Ala/Thr
2	13,997,380	MtrunA17Chr2g0295561	8° intron	Putative condensin II complex subunit H2	
2	18,946,103	MtrunA17Chr2g0301251	8° exon	Putative transcription factor ARF family	Ile/Val
2	31,530,957	MtrunA17Chr2g0308681	1° exon	Putative transcription factor C2H2 family	Glu/Gly
2	42,905,336		intergenic region		
3	4,199,596	MtrunA17Chr3R0023360	repeat region 4305	DTX_singleton_family2571	
3	35,488,554	MtrunA17Chr3g0113821	1° exon	Putative omega-hydroxypalmitate O-feruloyl transferase	Asn/Asp
3	42,791,235	MtrunA17Chr3g0123691	3° exon	Putative transcription factor Homobox-WOX family	Ala/Thr
3	45,584,895	MtrunA17Chr3g0127571	6° intron	CDPK-related kinase	
3	49,997,294	MtrunA17Chr3g0133511	2° exon	Putative Serpin family protein	Asn/Ser
4	5,957,228	MtrunA17Chr4g0007641	1°exon	Putative DNA (cytosine-5-)-methyltransferase	Arg/Cys
4	20,298,935	MtrunA17Chr4g0021701	3° exon	Putative lanthionine synthetase C, six-hairpin glycosidase-like superfamily	Arg/Lys
4	24,404,523	MtrunA17Chr4g0023841	2° intron	Putative mitochondrial import protein TIM15	
4	30,266,067	MtrunA17Chr4g0029431	1° exon	Putative fasciclin-like arabinogalactan protein, group A	Gly/Glu
4	36,226,410	MtrunA17Chr4g0037691	7° intron	Putative Zinc finger, RING/FYVE/PHD-type	
4	40,766,150	MtrunA17Chr4g0043781	4° exon	Putative sulfiredoxin	
4	49,322,710	MtrunA17Chr4g0055761	1° exon	Putative transcription factor AP2-EREBP family	Glu/Gly
4	50,443,296	MtrunA17Chr4g0057141	2° exon	Putative transcription factor WD40-like family	Asn/Asp
4	51,871,722	MtrunA17Chr4g0059061	4° intron	Putative poly (ADP-ribose) polymerase	
4	64,483,854	MtrunA17Chr4g0076731	3° exon	Putative transcription factor NAM family	Gly/Ser
5	5,313,443	MtrunA17Chr5g0401061	5° intron	Putative transcription factor MYB-HB-like family	
5	9,011,449	MtrunA17Chr5g0405891	1° exon	SKP1-like 2	Ala/Val
5	14,568,730	MtrunA17Chr5g0413331	10° intron	Putative ATP citrate synthase	
5	26,703,532	MtrunA17Chr5g0424671	3’ UTR region	Putative protein	
5	35,996,706	MtrunA17Chr5g0436081	4° exon	Putative transcription factor WD40-like family	Val/Ile
5	40,853,460	MtrunA17Chr5g0442821	6° intron	Putative tetratricopeptide-like helical domain superfamily	
5	43,013,859	MtrunA17Chr5g0446431	4° exon	Putative cystathionine beta-synthase	
6	1,337,680	MtrunA17Chr6g0450621	1° exon	Putative protein	
6	20,451,475	MtrunA17Chr6g0470341	1° intron	Putative DHBP synthase RibB-like alpha/beta domain superfamily	
6	41,555,486	MtrunA17Chr6g0487261	2° exon	beta-1,3-Glucanase 45	Pro/Leu
7	9,263,617	MtrunA17Chr7g0224051	4° exon	CLASSY-like 2 (putative chromatin remodelling factor)	
7	11,942,546	MtrunA17Chr7g0226791	8° intron	Putative transcription factor C2H2 family	
7	37,108,355	MtrunA17Chr7g0249241	1° exon	Putative pentatricopeptide repeat-containing protein	Thr/Ile
7	45,012,438	MtrunA17Chr7R0277390	repeat region 6960	Repeat_region6960.1 RLG_singleton_family450	
7	45,065,601	MtrunA17Chr7g0260161	3° exon	Putative protein Networked (NET), actin-binding (NAB)	
7	48,259,765	MtrunA17Chr7g0264841	1° intron	Putative Myb/SANT-like domain-containing protein	
7	48,472,569	MtrunA17Chr7g0265071	3’ UTR region	Putative protein	
7	49,340,664		intergenic region		
7	51,362,536	MtrunA17Chr7g0269361	1° exon	Putative pectinesterase	Glu/Gly
8	1,679,834	MtrunA17Chr8g0336451	9° intron	Putative MIF4G-like, type 3, initiation factor eIF-4 gamma	
8	7,132,036	MtrunA17Chr8g0343811	3° exon	Putative transcription initiation factor TFIID subunit 12	Pro/Ala
8	8,939,469	MtrunA17Chr8g0345971	1° exon	Putative tripeptidyl-peptidase II	Stop/Trp
8	13,478,170	MtrunA17Chr8g0351341	1° exon	Putative concanavalin A-like lectin/glucanase domain superfamily	
8	15,278,931	MtrunA17Chr8g0353481	2° exon	Putative ribosome recycling factor	Ser/Pro
8	34,140,083		intergenic region		
8	49,691,016	MtrunA17Chr8g0393341	1° exon	Pentatricopeptide repeat-containing protein/DYW domain-containing protein	Phe/Ser

Contr: Jemalong 2HA10-9; LF: late flowering plants; AA change: amino acid change found in the protein sequence

In the 2345 segregating line, among the 16 SNPs selected, two SNPs were located in introns, two in the 5’ UTR region, two in the intergenic regions and 10 in exons, seven of which resulting in amino acid changes in the corresponding protein sequences (Table 5).

**Table 5:** *M. truncatula* 2345 line selected SNPs

Chromosome	Position	Gene (*M. truncatul*a A17 r5.0 genome)	SNP position	Gene function	AA change WT/LF
1	4,186,225		intergenic region		
1	34,450,164	MtrunA17Chr1g0183781	4° exon	Plant U-box protein 6	
1	39,470,281	MtrunA17Chr1g0190201	1° exon	Pentatricopeptide repeat-containing protein	Gly/Arg
1	40,315,896	MtrunA17Chr1g0191211	2°exon	Putative protein	
1	45,201,433	MtrunA17Chr1g0198131	1° exon	Putative OTU domain, papain-like cysteine peptidase superfamily	
1	46,406,388	MtrunA17Chr1g0199691	4° exon	Putative phosphotransferase (phosphomutase)	Asp/Asn
1	46,672,493	MtrunA17Chr1g0200031	3° exon	Putative protein	Thr/Ala
1	56,557,638	MtrunA17Chr1g0213771	1° intron	Putative fungal lipase-like domain, alpha/Beta hydrolase	
2	1,793,509	MtrunA17Chr2g0279331	2° exon	Putative armadillo-like helical, splicing factor 3B subunit 1	Ile/Met
3	4,012,155		intergenic region		
5	29,119,518	MtrunA17Chr5g0427271	7° exon.	Putative Rho GTPase activation protein	Thr/Ala
5	29,119,525		7° exon.		Val/Ala
5	34,431,150	MtrunA17Chr5g0434281	5° intron	Putative phenylalanine–tRNA ligase	
6	14,509,266	MtrunA17Chr6g0465291	1° exon	Putative non-specific serine/threonine protein kinase	Gly/Cys
8	37,915,579	MtrunA17Chr8g0376321	5’ UTR region	Putative (H+)-transporting two-sector ATPase	
8	37,915,580		5’ UTR region		

LF: late flowering mutant (bulk of three plants); AA change: amino acid change found in the protein sequence

Two SNPs were common to both lines: the C/T changes in MtrunA17_Chr1g0191211 and in MtrunA17_Chr1g0200031 (Tables 4 and 5). In MtrunA17_Chr1g0200031, with 67% identity to the serine/threonine-kinase At4g25030, and coding for an uncharacterized protein component of chloroplast membrane, the SNP produced an amino acid change. In both cases, the alleles characterizing the LF individuals were opposite in the two lines, making it unlikely their involvement in the LF phenotype.

Many of the identified genes, 12 out of 56 total genes found in the two mutant lines, belonged to the TF family and different TF classes were represented (Tables 4 and 5). Other genes were involved in DNA methylation or remodelling (MtrunA17Chr4g0007641, MtrunA17Chr7g0224051 in *Mt* line 1739), RNA processing (MtrunA17Chr2g0279331 in *Mt* line 2345), protein synthesis or regulation (MtrunA17Chr6g0465291, MtrunA17Chr5g0434281 in *Mt* line 2345). All these gene families are involved in the regulation of plant growth and development, including flowering induction process.

### Gene ontology (GO) analysis

**Fig. 3:**
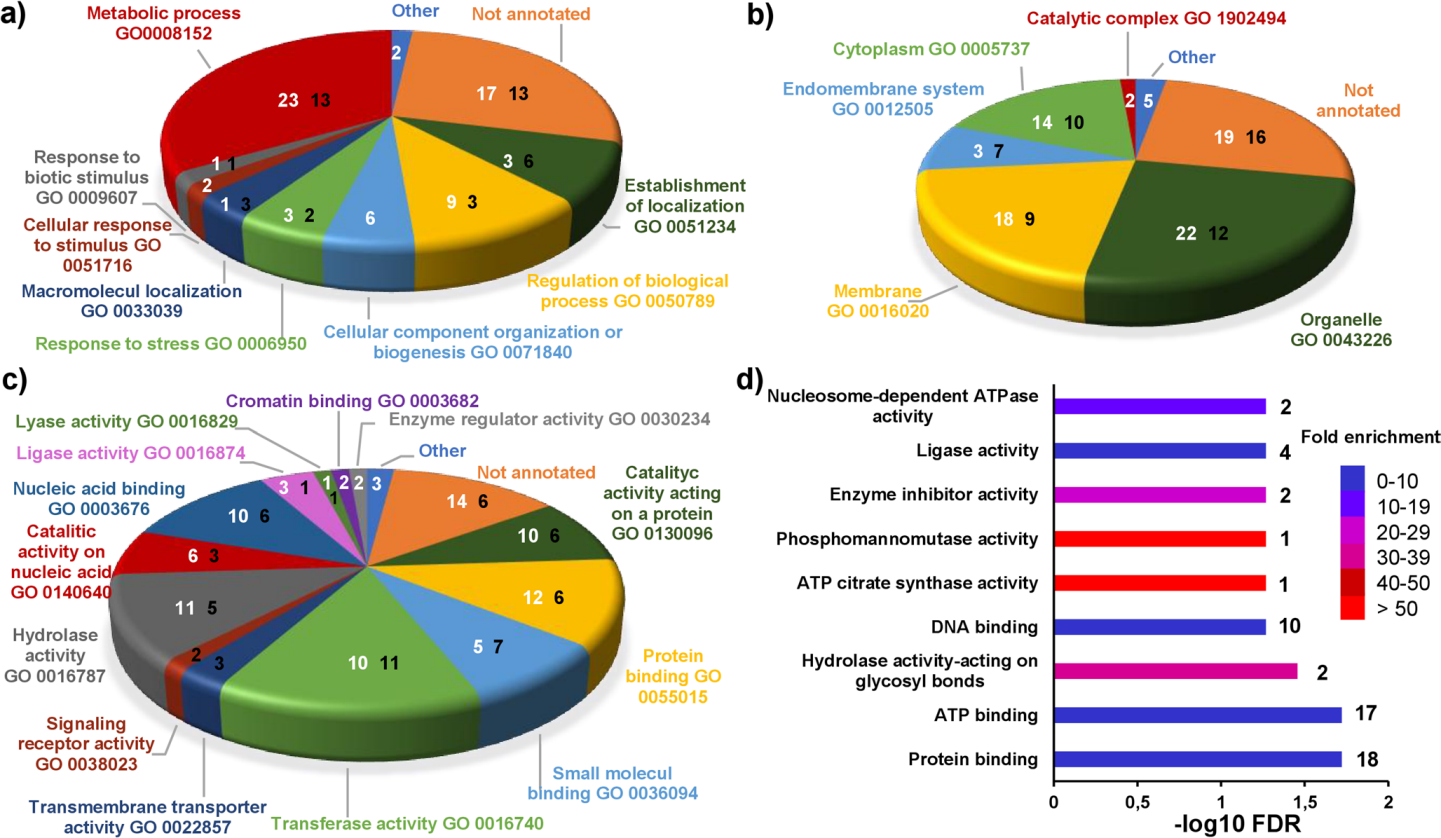
Gene ontology (GO) classification and enrichment analysis of the 91 genes carrying SNP in alfalfa EF-LF lines (35 genes) and in *M. truncatula* mutant lines (44 genes in *Mt* 1739 and 12 in *Mt* 2345). Level 3 GO terms for:** a** Biological Process (BP);** b** Cellular Component (CC);** c** Molecular Function (MF). Numbers in the GO term segments represents alfalfa (black) and *M. truncatula* (white) genes; each gene can be present in more than one GO term.** d** Significantly enriched GO terms for Molecular Function category (FDR < 0.06). Numbers represent the genes belonging to each GO terms

To gain additional insight into genes potentially involved in the regulation of flowering, the genes presenting significant SNPs in alfalfa (Online Resource 5) and *M. truncatula* (Tables 4 and 5) were functionally classified according to Blast2GO (https://www.ebi.ac.uk/QuickGO). Excluding redundancy, 91 genes were analysed, 35 from alfalfa and 56 from *M. truncatula*. The analysed genes were distributed in the three main GO terms, i.e. Biological Processes (BP), Cellular Component (CC) and Molecular Functions (MF) (Fig. 3a, b and c, respectively and Online Resource 9). The MFs Transferase activity and Transmembrane transporter activity (GO 0016740 and GO 0022857) (Fig. 3c) appeared consistent with the CCs Membrane and Endomembrane system (GO 0016020 and GO 0012505) (Fig. 3b) in indicating a flux of signals through cellular compartments involving also organelles (GO 0043226). The presence of MFs Binding of protein (GO 0055015), nucleic acid (GO 0003676) and small molecule (GO 0036094) (Fig. 3c) agreed with the BP Regulation of biological process (GO 0050789) in Fig. 3a. Both alfalfa and *Mt* genes were present in most of the BP, CC and MF GO term subdivisions (Fig. 3a, b and c, respectively) but no common gene putatively involved in flowering time control was found between the two *Medicago* species. These results indicate a large overlap of the gene classes involved in the trait in the two species but also suggest a diversification in the gene combinations actually set up by alfalfa and *M. truncatula* in the divergent earliness profile.

A GO enrichment analysis was also performed using the ShinyGO v0.81 tool; a significant GO enrichment was found only in MF category. The 91 genes were grouped in nine significant (FDR < 0.06) GO terms (Fig. 3d). The phosphomannomutase activity (GO 0004615), ATP citrate synthase activity (GO 0003878) and hydrolase activity-acting on glycosyl bonds (GO 0016798) were the GO terms showing the greater enrichment.

Concerning alfalfa synthetics with divergent stem morphology, the 11 identified genes were distributed in the three main GO terms BP, CC and MF (Online Resource 10 a, b and c, respectively and Online Resource 9); the small number of genes made it impossible to apply the GO enrichment analysis.

### Protein structural disruption analysis

Thirty-four total nsSNPs, resulting in amino acid changes for the encoded protein, were considered: 6 from *M. sativa* ‘lines’ (five for flowering time and one for stem morphology), 21 derived from *M. truncatula* line 1739 and 7 from line 2345.

For two genes (MtrunA17Chr3g0120731 in alfalfa lines divergent for flowering time and MtrunA17Chr8g0345971 in *Mt* 1739 line), the SNP implied the formation of a STOP codon ans so were considered a-priori disruptive of the protein structure and not further analysed.

DUET ΔΔG values, integrating the other metrics mCSM and SDM using a non-linear model (Pires et al. 2014a), were mainly considered in the analysis (Online Resource 11).

In alfalfa lines divergent for flowering time, the DUET ΔΔG values were positive (MtrunA17Chr4g0075951, MtrunA17Chr2g0292681), around zero (MtrunA17Chr3g0084091) or weakly negative (MtrunA17Chr7g0240901) suggesting non-disruptive changes in the protein structures and the maintenance of a functional role in the alternative proteins present in LF lines. Similarly, in the alfalfa synthetics divergent for internode length the positive DUET ΔΔG value of the putative protein kinase RLK-Pelle MtrunA17_Chr3g0128551 suggested the conservation of the alternative protein structure present in SIsyn and its functional role (Online Resource 11).

In *M. truncatula* lines, three genes (MtrunA17Chr1g0158991, the MtrunA17Chr8g0393341 in *Mt* 1739 line and the MtrunA17Chr6g465291 in *Mt* 2345 line) resulted in DUET ΔΔG negative values strongly indicating potential structural disruption (− 1.893, − 1.984 and − 1.798 kcal/mol, respectively). Other four genes in *Mt* 1739 line (MtrunA17Chr1g0200031, MtrunA17Chr3g0123691, MtrunA17Chr4g0029431 and MtrunA17Chr4g0076731) showed DUET ΔΔG values between − 0.5 and − 1 kcal/mol, suggesting a potential for structurally disruptive changes. All the other DUET ΔΔG values in *Mt* lines were suggestive of protein structure conservation in LF individuals within each line (Online Resource 11).

## Discussion

Flowering time and dynamics and internode length are traits related to quality in alfalfa. In fact, crop developmental stage at harvest is a major factor affecting the forage intake, digestibility and the protein concentration (Palmonari et al. 2014) and a greater proportion of leaves, increased stem digestibility or both are indicated as significant traits controlling alfalfa nutritive value (Julier et al. 2000; Jung and Lamb 2006; Lorenzo et al. 2020). In recent papers alfalfa genetic variation has been used to identify quality-related traits (Lin et al. 2025; Xu et al. 2025a, b). Genetic variation (SNPs) differentiating the alfalfa lines/synthetics obtained by divergent selections for these traits allowed us to identify gene classes and specific genes implied in the control of these same traits and then potentially interesting for the improvement of forage quality in alfalfa.

### Regulation of flowering time and dynamics

None of the main genes involved in flowering control previously identified in *Medicago* spp. were found to carry SNPs in our plant materials except the phytochrome *MtPHYB* (MtrunA17Chr2g0294361) in alfalfa line EF5-LF5 comparison where the two SNPs present did not result in amino acid change (Online Resource 5).

However, many of the gene classes identified in our analysis have been shown to be involved in the complex regulatory network integrating endogenous and exogenous signals controlling flowering time in dicot herbaceous species.

### Transcription factors

In our alfalfa and *Mt* TILLING lines dataset, TFs were the most represented class (19.8%) with 18 out of the 91 total genes (Tables 2, 4 and 5).

Among the TFs, the putative TF interactor and regulator CCHC(Zn) MtrunA17Chr7g0240901 found in alfalfa EF1-LF1 and EF6-LF6 comparisons (Table 2) had 70% identity with the nuclear 5’-3’ exoribonuclease At1g75660 involved in RNA turnover. The At1g75660 homologs in tomato, Solyc04g081280 and Solyc12g089280, were active in the retrograde signaling i.e. in the regulation of nuclear gene expression in response to the developmental stage and functional state of the plastids (Pesaresi et al. 2014).

In the homozygous LF *Mt*1739 line, the C2H2 zinc finger protein (ZPF) MtrunA17Chr2g0308681 gene presented a non-synonymous SNP (Table 4). In *Arabidopsis*, the C2H2 ZFP EARLY FLOWERING 6 (*AtELF6*, At5g04240) and RELATIVE OF EARLY FLOWERING 6 (*AtREF6*, At3g48430) played significant roles in regulating floral transition as FLC (Floral Locus C) repressor (*AtREF6*) or as an upstream repressor in the photoperiodic flowering pathway (*AtELF6*) issuing in LF and EF phenotypes, respectively (Noh et al. 2004). Two genes encoding *Arabidopsis* C2H2 ZFP TFs, *AtZP1*(At4g17810) and *AtZFP8* (At2g41940), are involved in floral meristem induction (Hu et al. 2023) acting redundantly to repress the floral homeotic genes APETALA 3 (*AtAP3*), PISTILLATA (*AtPI*) and AGAMOUS (*AtAG*) in leaves.

The putative TF NAM family *Mt*1739 MtrunA17Chr4g0076731 (Table 4) belongs to plant-specific NAC TF family involved in the regulation of the transition from vegetative to reproductive growth in many plant species (Liu et al. 2023). MtrunA17Chr4g0076731 showed 75% identity with *Arabidopsis* ANAC52 (Ling et al. 2017) corresponding to the locus At2g02450. In *Arabidopsis* the overexpression of At2g2450 (*AtLOV1*) in transgenic lines caused a long vegetative phenotype *via* downregulation of the floral promoter *CONSTANS* (CO) while the loss of function resulted in a slightly early-flowering phenotype (Yoo et al. 2007). On the contrary, the MtrunA17Chr4g0076731 encoded protein, showing a potential for structural and functional disruptive changes (Online Resource 11), resulted in LF phenotype in *Mt*1739 line. As the central role of *AtCO* and *CONSTANS*-LIKE (*AtCOL*) genes in the photoperiod response mechanism was not conserved in *M. truncatula* and in other legumes (Wong et al.^ 2014^), a different role for MtrunA17Chr4g0076731 should be hypothesized in this species.

The putative TF APETALA2/Ethylene Responsive Factor (AP2/ERF) *Mt*1739 MtrunA17Chr4g0055761 (Table 4) belongs to a class of regulatory proteins involved in the control of primary and secondary metabolism, growth and developmental programs, as well as responses to environmental stimuli (Licausi et al. 2013). In *Arabidopsis*, the AP2/ERF AINTEGUMENTA is involved in the regulation of flower development (Krizek 2009).

The two *Mt*1739 genes MtrunA17Chr4g0057141 and MtrunA17Chr5g0436081 (Table 4) belong to the WD40 TF family WDR proteins (WD40 protein domain, or beta-transducin repeat-WDRs) known to act as hubs for protein-protein or protein-nucleic acid interactions (Jain and Pandey 2018). In *Arabidopsis*, a mutation in the CONSTITUTIVELY PHOTOMORPHOGENIC1 (*AtCOP1*) WD40 protein proved to be involved in early flowering phenotype (Yu et al. 2023).

The *Mt*1739 putative TF initiation factor (TFIID) MtrunA17Chr8g0343811 is part of TFIID subcomplex for RNA polymerase transcription initiation consisting of TATA-box binding protein (TBP) and TBP associated factors, including TAF12. In *Arabidopsis*, TAF12 was found to take part in the unfolded protein response, a versatile stress sensing mechanism to respond to a wide range of biotic and abiotic stimuli (Kim et al. 2022).

### Protein involved in DNA/RNA modification and remodelling

In plant genomes, DNA methylation, relying on specific DNA methyltransferases, is known to be involved in flowering regulation (Shi et al. 2023). In our dataset, the *Mt*1739 MtrunA17Chr4g0007641 gene (Table 4) encoding for a DNA (cytosine-5-)-methyltransferase was interested by a nsSNP and present in DNA Binding GO enriched class (Fig. 3d). *Arabidopsis* mutant plants in methyltransferase cDNA *AtMETI* showed decreased methylation associated to phenotypic and developmental abnormalities, including an altered flowering time (Finnegan et al. 1998) and the *Atfwa* mutant displayed late flowering phenotype associated with hypomethylation of *AtFWA* gene (Soppe et al. 2000). The variation in the flowering time among 27 *Arabidopsis* populations was significantly correlated with methylation of the coding regions of six genes belonging to the complex pathways involved in flower induction (Xie et al. 2024).

Pentatricopeptide repeat-containing (PPR) proteins are sequence-specific RNA-binding proteins involved in plant organellar (plastids and mitochondria) RNA processing as splicing, cleavage and translational initiation, controlling organellar gene expression (Barkan 2014). The tetratricopeptide repeat protein MtrunA17Chr2g0292681, carrying a nsSNP, was found in in *Ms* EF2-LF2, EF4-LF4 and EF6-LF6 comparisons (Table 2); besides, four PPR proteins were identified in *Mt* TILLING lines 1739 and 2345 (Tables 4 and 5), three of which - MtrunA17Chr7g0249241 and MtrunA17Chr8g0393341 in *Mt*1739 and MtrunA17_Chr1g0190201 in *Mt*2345 - showing an amino acid change. For MtrunA17Chr8g0393341 gene, the structural analysis strongly suggested an alteration of protein three-dimensional structure (Online Resource 11). In *Arabidopsis*, a mutant in the PPR gene At1g15480, involved in mitochondrial RNA editing, exhibited early-flowering phenotype due to a strong downregulation of the floral repressor FLC (Emami and Kempken 2019) and the leaf-specific PPR gene *AtC401* (At5g21222) with protein kinase activity form a distinct group displaying a circadian rhythmic expression (Oguchi et al. 2004). These evidences make the PPR proteins suitable candidate genes for flowering time regulation in *Medicago* spp.

### Sugar and lipid metabolisms

The plant endogenous nutritional status is an essential factor controlling flowering; sugars produced in the leaves, in particular sucrose, transferred to the shoot apical meristem act as signalling molecules regulating flower induction (Corbesier et al. 1998). MtrunA17_Chr1g0199691 gene carrying an amino acid change in *Mt*2345 line (Table 5) encodes for a putative phosphomannomutase catalysing the interconversion from mannose-6-phosphate to mannose-1-phosphate necessary for the synthesis of GDP-mannose, an important precursor of the antioxidant ascorbic acid (AsA) in higher plants (Qian et al. 2007). Due to its essential function as co-factor for the biosynthesis of GA and ABA, AsA influences both the endogenous level and signalling of these hormones thus affecting developmental flowering and senescence in a presumably photoperiod- and/or circadian rhythm-dependent manner (Kotchoni et al. 2009). These evidences make MtrunA17_Chr1g0199691 a suitable candidate gene for regulation of flowering time in *Medicago* spp.

Anther-specific β-1-3 glucanases are responsible for the degradation of the cell wall callose surrounding the tetrad thus releasing individual microspores into anther locule; controlled degradation of callose deposits in plasmodesmata interconnecting cytoplasm and endoplasmic reticulum of adjacent cells is relevant for axillary bud growth and shoot apical meristem determination. In our dataset, two β-1-3 glucanase genes were found: MtrunA17Chr8g0378621 in alfalfa with a SNP in intron (Online Resource 5) and MtrunA17Chr6g0487261 (Table 4) carrying an amino acid change in *Mt*1739 line. Two *Arabidopsis* pollen/stamen-specific β-1-3 glucanases (At5g20390 and At5g64790) were involved in pollen development and maturation (Doxey et al. 2007) while the association of callose accumulation in flowers with female sterility in alfalfa was suggested by Capomaccio et al. (2009).

Other genes involved in sugar metabolism and cell energy balance were the alfalfa sucrose synthase 2 MtrunA17_Chr7g0269581 (Online Resources 5) and the ATP citrate synthase MtrunA17Chr5g0413331 in *Mt*1739 line (Table 4) both carrying a SNP in intron. In addition, an ATPase gene (MtrunA17_Chr8g0376321) with two SNP in 5’ UTR region was found in *Mt*2345 TILLING line (Table 5).

Lipid metabolism plays key roles in plant reproductive development, especially in anther cuticle and pollen wall development (Shi et al. 2015), anther dehiscence and pollen maturation (Ishiguro et al. 2001; Xu et al. 2025a, b), embryo and female gametophyte development (Kim and Huang 2004), sex determination, spikelet development, and flowering (Acosta et al. 2009). The long-chain-fatty-acid-CoA ligase MtrunA17_Chr3g0120731 - carrying a nsSNP in alfalfa lines (Table 2) - and the lipase MtrunA17_Chr1g0213771 in *Mt*2345 lines (Table 5) with a SNP in intron were found in our material. MtrunA17Chr3g0120731 had 72% identity with the long-chain acyl-coenzyme A (CoA) synthetase (LACS) *At*LACS8 (At2g04350) mainly expressed in leaves and flowers in *Arabidopsis*). *At*LACS8 is localized in the endoplasmic reticulum, where membrane/cuticular lipids and triacylglycerols are produced, with the function of activating *de novo* synthesized long-chain fatty acids in plastids (Zhao et al. 2019). MtrunA17Chr3g0120731 SNP implied a change from an amino acid to a STOP codon (Online Resource 11), strongly suggesting a loss-of-function protein in EF alfalfa lines.

### Response to environmental conditions (light and temperature)

The *M. sativa* lines with divergent earliness were likely to imply a variation in light and temperature perception and in the relative adaptive responses. Moreover, the six EF-LF couples derived from S_2_ parents differing in photoperiod sensing (non-dormant and fall dormant, Online Resource 1). In fact, there were evidences that organelles and particularly chloroplasts, involved in adaptation to changing light parameters and temperature, play an important role in determining the EF and LF profile in our plant materials. Primarily, the Organelle GO 0043226 accounted for 12 and 22 among the 91 genes carrying SNPs in alfalfa EF-LF lines and *Mt* TILLING lines, respectively, in GO terms for Cellular Component (Fig. 3b). Secondly, the tetratricopeptide repeat protein MtrunA17Chr2g0292681 in alfalfa and the four pentatricopeptide repeat-containing (PPR) proteins in *Mt* lines discussed in paragraph 1.2 are specifically committed to the control of organellar gene expression. Thirdly, the two alfalfa genes MtrunA17Chr4g0075951 and MtrunA17Chr4g0076431 (Table 2) coded for a putative WEB family protein showing 44% and 46%, respectively with At1g12150 related to AtPMI2 (At1g66840). In *Arabidopsis*, WEB1 (At2g26570) and *AtPMI2* form a cytosolic protein complex that cooperatively maintains the velocity of chloroplast movement in response to different light conditions *via* chloroplast actin filament regulation (Kodama et al. 2010). Finally, the *Mt*2345 phosphomannomutase MtrunA17Chr1g0199691 gene, discussed in paragraph 1.3, resulted localized in chloroplast stroma and MtrunA17Chr1g0200031, one of the two SNPs common to both *Mt* lines, is a component of the chloroplast endomembrane system.

### Stem morphology

Plant height, mainly determined by the number of stem nodes and internode length, represents an important agronomic trait influencing both biomass yield and quality ( Xu et al. 2023). In the case of alfalfa the improvement of the leaf-to-stem ratio by increasing the number of internodes carrying leaves/branches and decreasing their average length, was the objective of our breeding program (Scotti et al. 2007; Pecetti et al. 2017). Despite the identification, cloning and functional characterization of many genes related to internode elongation and plant height in diverse crop species mainly related to hormone biosynthesis, metabolism and signalling pathways (Jing et al. 2023), few studies on mechanism of stem elongation in *Medicago* spp. have been reported. Dwarf, prostrate and bushy mutants of *M. truncatula* and alfalfa have been characterised, the underlying genes have been cloned, and in some cases their functions have been studied (Jaudal et al. 2018; Guo et al. 2020b; Zhang et al. 2020; Zheng et al. 2022). In our alfalfa populations, we identified eleven genes potentially involved in the divergent stem morphology among which protein kinases represent the most important gene class (Table 3).

### Protein kinases

MtrunA17Chr3g0128551 gene, carrying a nsSNP differentiating LIsyn/SIsyn alfalfa synthetics (Table 3), is a cell surface-localized receptor-like kinase (RLKs). RLK genes act as receptors of extracellular signals and transducer to intracellular signaling pathways. In melon, mutant plants in CmSi gene, encoding an ERECTA-like receptor kinase, showed short internodes phenotype. CmsI exhibited high expression in stem vascular bundle during internode elongation, positive correlation between its expression level and stem length and promotion of stem elongation in *Arabidopsis* and cucumber when over-expressed (Yang et al. 2020). The role of ERECTA family (ER) receptor-like kinases on plant growth, morphogenesis and development has been investigated in recent years (reviewed by Jiang et al. 2022; Liu et al. 2024). The inactivation of RLKs genes determined several developmental modifications, including short internodes and reduction of plant height (Shpak et al. 2004; Yang et al. 2020; Xin et al. 2022; Xu et al. 2023; Zhao et al. 2023). These evidences make the MtrunA17Chr3g0128551 a suitable candidate gene for stem morphology regulation in *Medicago* spp.

## Conclusions

The alfalfa narrow-based ‘lines’ genotyped via GBS analysis proved to be effective in identifying a genetic variation (SNPs) underlying their divergent profiles for complex traits as time/dynamics of flowering and stem morphology. In fact, most of the genes found carrying significant SNPs differentiating the divergent ‘lines’ belonged to gene classes known for their involvement in flowering and/or stem length control in herbaceous dicot species. The divergent selection applied in the selfing phase was then successful in concentrating genes and gene combinations controlling the traits of interest in the S_2_ selected parents and in the successive 2-constituent synthetics (‘lines’) as proved by Rotili for alfalfa yield (1976). Consistent with the function of integrating and channeling environmental and endogenous signals towards stem growth and flowering transition, the putative genes found belonged to different regulation levels from DNA/RNA/protein modification and remodelling and TFs to biosynthetic processes pertaining to sugar and lipid metabolism. The functional relationship between nucleus and organelles, in particular chloroplasts, emerged as a key point in defining the different earliness profile of the alfalfa ‘lines’.

In this study, putative candidate genes relevant to key quality-related traits were identified. These genes represent starting points for future functional characterization able to validate their direct role in traits object of the selection in *Medicago* spp. thus opening new strategies to improve alfalfa forage nutritive value through the manipulation of flowering processes and stem morphology. For this purpose, the emerging application of genome-editing technology, in particular the CRISPR/Cas9 system, represents a versatile and valuable tool for functional genomics studies on nutritional-related traits (Wolabu et al. 2024). The functional characterization of the most promising genes is currently underway. Furthermore, selected significant and validated SNP markers may be useful for marker-assisted selection to develop elite alfalfa varieties with enhanced nutritional value.

## Supplementary Information

Below is the link to the electronic supplementary material.

Online Resource

## Data Availability

The data supporting the conclusions of this study are included in this published article as Additional files. All results produced in GBS analysis have been deposited in Figshare database under the accession number 10.6084/m9.figshare.29684315.
