# Supplementary material for: Alfalfa and M. truncatula TILLING lines divergent for nutritive value-related traits enable the identification of novel candidate genes by genotyping by Sequencing approach
Source: Physiol Mol Biol Plants. 2026 May 26;32(6):1243–60. doi: 10.1007/s12298-026-01750-2 (PMC13291374; doi:10.1007/s12298-026-01750-2)
Supplement: Supplementary file 1 — Online Resource [file 12298_2026_1750_MOESM1_ESM.pdf]

Alfalfa and *M. truncatula* TILLING lines divergent for quality-related traits enable the identification of novel candidate genes by Genotyping by Sequencing approach.

Online Resource 1: Medicago sativa early (EF) and late (LF) flowering 'lines' analysed.

M. sativa 'line'	Generation	Parental cross (SHs)	No. of plants bulked
EF1	$2S_2$syn3	1x2	5
EF2	$2S_2$syn3	1x5	9
EF3	$2S_2$syn3	1x7	6
EF4	$2S_2$syn3	2x5	8
EF5	$2S_2$syn3	2x7	5
EF6	$2S_2$syn3	5x7	8
EFsyn	$4S_2$syn3	4-constituent syn	15
LF1	$2S_2$syn3	1x2	4
LF2	$2S_2$syn3	1x5	6
LF3	$2S_2$syn3	1x7	8
LF4	$2S_2$syn3	2x5	7
LF5	$2S_2$syn3	2x7	7
LF6	$2S_2$syn3	5x7	8
LFsyn	$4S_2$syn3	4-constituent syn	10

Parents 1 and 2: Mediterranean origin; no fall dormancy.
Parents 5 and 7: continental origin; fall dormancy.

Online Resource 2: *M. sativa* 'lines' with divergent earliness (early, EF/late, LF flowering). DMY and parameters estimating earliness at single plant basis (means of harvests 1-5 for DMY and 2-5 for earliness traits): ANOVA.

Sources of variation	df	DMY (g)		Stem height (cm)		Stem reproductive part (%)		No. of reproductive nodes		Earliness index	
		F	Sign	F	Sign	F	Sign	F	Sign	F	Sign
Flowerig time (EF/LF)	1	10.74	0.002***	0.01	0.91 ns	88.24	****	74.38	****	81.96	****
Cross	6	6.76	****	14.49	****	3.91	0.004***	4.28	0.002***	5.47	****
Block	3	13.26	****	4.93	0.005***	11.40	****	12.42	****	14.52	****
Fl.time x cross	6	3.28	0.01*	6.29	****	3.93	0.004***	2.33	0.05 ‡	4.57	0.001***
EF vs LF in 1x2[1]	1	12.97	****	5.56	0.02 *	1.65	0.21 ns	3.38	0.07 ns	1.12	0.30 ns
EF vs LF in 1x5[1]	1	0.27	0.61 ns	10.16	0.003***	33.28	****	16.38	****	31.24	****
EF vs LF in 1x7[1]	1	0.00	0.98 ns	1.40	0.24 ns	7.50	0.009**	4.69	0.04 *	10.43	0.002***
EF vs LF in 2x5[1]	1	0.71	0.40 ns	3.82	0.06 ns	37.54	****	37.40	****	37.15	****
EF vs LF in 2x7[1]	1	11.17	0.002***	8.26	0.006**	2.43	0.13 ns	4.63	0.04 *	1.11	0.30 ns
EF vs LF in 5x7[1]	1	0.04	0.83 ns	7.30	0.01*	6.94	0.01 *	6.91	0.01 *	3.95	0.05 ‡
EF vs LF in syn[1]	1	5.22	0.03 *	1.26	0.27 ns	22.48	****	14.99	****	24.37	****
Error	39										

[1]Comparisons between early and late flowering time within each cross by linear contrasts.

Significant at: ‡ 0.06<P>0.05; * P<0.05; ** P<0.01; *** P<0.005; **** P<0.001; ns, not significant.

Online Resource 3: *M. sativa* 'lines' with divergent earliness (early/late flowering): DMY and parameters estimating earliness at single plant basis (means of harvests 1-5 for DMY and 2-5 for earliness traits).

Flowering time	*Ms* 'line' syn3 generation	Cross	DMY (g)	Stem height (cm)	Stem reproductive part (%)	No. of reproductive nodes	Earliness index
Early	EF1	1x2	1.61****[1]	53.68 *[1]	3.30 ns[1]	2.31 ns[1]	10.04 ns[1]
	EF2	1x5	1.71	53.24 ***	6.24 ****	2.69 ****	17.77 ****
	EF3	1x7	1.59	45.76	3.04 **	2.18 *	10.42 ***
	EF4	2x5	2.03	55.82	6.17 ****	2.88 ****	17.80 ****
	EF5	2x7	1.45 ***	48.93 **	3.20	2.36 *	9.54
	EF6	5x7	1.45	49.23 **	2.81 *	1.91 *	6.60 ‡
	EFsyn	4syn	2.00 *	55.36	5.71 ****	2.93 ****	18.38 ****
Late	LF1	1x2	2.35	58.83	2.20	1.85	7.49
	LF2	1x5	1.82	46.29	1.26	1.67	4.32
	LF3	1x7	1.58	48.33	0.68	1.64	2.65
	LF4	2x5	1.86	51.56	0.88	1.34	3.13
	LF5	2x7	2.14	55.20	1.86	1.82	7.00
	LF6	5x7	1.40	43.33	0.54	1.25	1.81
	LFsyn	4syn	2.47	57.81	1.61	1.95	6.50

[1]Comparisons between early and late flowering time within each cross by linear contrasts. Significant at: ‡ 0.06<P>0.05; * P<0.05; ** P<0.01; *** P<0.005; **** P<0.001.

Online Resource 4. *M. sativa* synthetics with divergent stem morphology (long, LI/short, SI internode length). DMY and parameters estimating stem morphology at single plant basis (means of harvests 2-4 of the sowing year): ANOVA.

Sources of variation	df	DMY (g)		Stem height (cm)		No. of vegetative internodes		Average vegetative internode length (cm)		Stem reproductive part (%)		No. of reproductive nodes		Earliness index	
		F	Sign	F	Sign	F	Sign	F	Sign	F	Sign				
Stem morphology[1] (SI/LI)	1	0.25	0.63 ns	88.69	***	12.11	*	56.69	***	15.79	0.03 *	40.33	0.008 **	25.11	0.02 *
Block	3	0.37	0.78 ns	7.12	0.01 *	1.60	0.26 ns	2.26	0.16 ns	2.86	0.10 ns	0.60	0.63 ns	0.44	0.73 ns
St.mor. x bl	3	2.26	0.16 ns	0.51	0.69 ns	3.33	0.08 ns	2.50	0.13 ns	8.72	0.007 **	2.49	0.13 ns	1.47	0.29 ns
Error	8														

[1]Stem morphology was tested using the interaction stem morphology x block as error term.
Significant at: * P<0.05; ** P<0.01; *** P<0.005; **** P<0.001; ns, not significant.

Online Resource 5: List of alfalfa SNPs identified in the comparisons of LF-EF alfalfa lines vs the corresponding EFSyn and LFSyn. Reference allele: allele found in Alfalfa genome; Alternative allele: allele found in sequenced plants; EF: early flowering lines bulk; LF: late flowering lines bulk; EFSyn: early flowering synthetic bulk; LFSyn: late flowering synthetic bulk; AA change: amino acid change found in protein. Number in columns represent the percentage of alleles differing from reference allele (1=100% difference).

Chromosome	position	Reference allele	Alternative allele	EF 1	LF 1	EFSyn	LFSyn	Gene name (*M. truncatula* A17 r5.0 genome)	SNP position	Gene function	AA change EF/LF
chr1_1	29345093	A	G	0	1	0	0,99	MtrunA17Chr1g0191161	4° exon	Putative WIYLD domain-containing protein	
chr1_1	43454407	C	T	0	1	0,06	0,94	MtrunA17Chr1g0176251	1° intron	Putative homogentisate solanesyltransferase	
chr3_1	89864578	C	G	0	1	0	0,93	MtrunA17Chr3g0144041	2° exon	Putative EH domain, EF-hand domain, EF-hand domain pair protein	His/Gln
chr3_1	90104449	C	T	1	0	0,98	0	MtrunA17Chr3g0144181	10° exon	Putative protein S-acyltransferase	
chr3_1	91883134	A	C	0	1	0	1	MtrunA17Chr3g0120731	4° exon	Putative long-chain-fatty-acid--CoA ligase	Stop/Glu
chr4_1	5593123	T	C	0	1	0	1	no alignement found			
chr4_1	7284752	G	A	1	0	1	0	MtrunA17Chr4g0036011	1° exon	Putative arginine decarboxylase	Ser/Gly
chr4_1	36184455	T	A	1	0	1	0	MtrunA17Chr4g0026411	9° intron	Putative protein	
chr4_1	74648725	A	C	1	0	1	0		intergenic region		
chr4_1	76319199	G	A	1	0	1	0	MtrunA17Chr3g0084091	5° exon	Putative EamA domain-containing protein	Leu/Ser
chr6_1	14867828	C	G	1	0	0,94	0	no alignement found			
chr7_2	26590611	G	A	1	0	1	0	MtrunA17Chr4g0077061		Putative RNA helicase	
chr7_2	32237060	C	G	0	1	0	1	MtrunA17Chr7g0240901	2° exon	Putative transcription factor interactor and regulator CCHC(Zn) family	Gln/Glu
chr8_1	50421996	A	G	0	1	0	1		intergenic region		
chr8_1	89476522	A	G	0	0,98	0,02	1	MtrunA17Chr4g0075951<br>MtrunA17Chr4g0076431	1° exon<br>2° exon	Putative WEB family protein	Thr/Ala<br>Thr/Ala

Chromosome	position	Reference allele	Alternative allele	EF2	LF2	EFsyn	LFsyn	Gene name (*M. truncatula* A17 r5.0 genome)	SNP position	Gene function	AA change
chr1_1	29345093	A	G	0	1	0	0,99	MtrunA17Chr1g0191161	4° exon	Putative WIYLD domain-containing protein	

chr1_1	51959447	T	C	0	1	0	0,96	MtrunA17Chr1g0183871	4° intron	Putative P-loop containing nucleoside triphosphate hydrolase	
chr1_1	58697761	C	T	0	1	0	1	MtrunA17Chr1g0190061	4° exon	Putative protein	Thr/Ile
chr1_1	69857191	T	G	0	1	0	1		intergenic region		
chr2_1	17246013	G	A	1	0	1	0	MtrunA17Chr2g0292681	4° exon	Putative tetratricopeptide repeat protein POLLENLESS 3/SULFUR DEFICIENCY-INDUCED 1	Thr/Ala
chr2_1	68555885	A	G	1	0	0,98	0	MtrunA17Chr2g0323941	11° exon	Putative non-specific protein-tyrosine kinase RLK-Pelle-LRR-V family	
chr4_1	48495396	C	T	1	0	1	0		intergenic region		
chr4_1	52496875	T	G	1	0	1	0	MtrunA17Chr4g0040511	11° intron	Putative transcription factor & chromatin remodeling DDT family	
chr4_1	52496876	G	A	1	0	1	0				
chr4_1	52496895	T	C	1	0	1	0				
chr4_1	61270806	C	T	0	1	0	1	MtrunA17Chr4g0052341	3'UTR region	Putative DNA helicase chromatin regulator PHD family	
chr4_1	76319199	G	A	1	0	1	0	MtrunA17Chr3g0084091	5° exon	Putative EamA domain-containing protein	Leu/Ser
chr6_1	2987373	C	T	1	0	1	0	MtrunA17Chr6g0450571	5'UTR region	Putative protein kinase STE	
chr7_2	48203985	A	T	0	0,96	0	0,94	MtrunA17Chr7g0237821	1° exon	Putative protein kinase RLK-Pelle-LRR-XI-1 family	Asp/Val
chr7_2	65828176	A	G	0,9	0	1	0	MtrunA17Chr7g0252141	3° intron	Putative transcription factor C2H2 family	
chr7_2	75842177	A	G	0	1	0	1	no alignement found			
chr8_1	7018471	C	G	0	1	0	1		intergenic region		
chr8_1	8489769	G	A	0	1	0	1	MtrunA17Chr8g0346601	2° exon	Putative tetratricopeptide-like helical domain superfamily	
chr8_1	8489778	C	T	0	1	0	1				

Chromosome	position	Reference allele	Alternative allele	EF3	LF3	EFsyn	LFsyn	Gene name (*M. truncatula* A17 r5.0 genome)	SNP position	Gene function	AA change
chr1_1	69857191	T	G	0	1	0	1		intergenic region		
chr2_1	3729299	G	C	1	0	0,92	0		intergenic region		

chr3_1	91883134	A	C	0	0,98	0	1	MtrunA17Chr3g0120731	4° exon	Putative long-chain-fatty-acid--CoA ligase	Stop/Phe
chr4_1	75590311	T	G	0	1	0	1	MtrunA17Chr8g0388131	2° exon	Putative nucleoside-diphosphate kinase	Ser/Ala
chr5_2	68493163	A	G	0	1	0	1	MtrunA17Chr5g0427261	3° intron	Putative protein	
chr5_2	68493216	T	C	0	1	0	1				
chr6_1	81846377	A	G	1	0	0,99	0	no alignement found			
chr7_2	78712665	T	C	1	0	1	0	MtrunA17Chr7g0264521	9° exon	Putative vesicle-fusing ATPase	
chr8_1	89476522	A	G	0	0,98	0,02	1	MtrunA17Chr4g0075951 MtrunA17Chr4g0076431	1° exon 2° exon	Putative WEB family protein	Thr/Ala Thr/Ala

Chromosome	position	Reference allele	Alternative allele	EF4	LF4	EFsyn	LFsyn	Gene name (*M. truncatula* A17 r5.0 genome)	SNP position	Gene function	AA change
chr1_1	35205946	C	G	0	1	0,083	0,93	MtrunA17_Chr1g0171471	3° exon	Putative glycerol-3-phosphate dehydrogenase	
chr1_1	64738449	C	T	1	0	1	0	MtrunA17_Chr1g0198461	3° exon	Putative R3H domain, SUZ domain, R3H domain superfamily	
chr1_1	69857191	T	G	0	1	0	1		intergenic region		
chr2_1	17246013	G	A	1	0	1	0	MtrunA17_Chr2g0292681	4° exon	Putative tetratricopeptide repeat protein POLLENLESS 3/ SULPHUR DEFICIENCY INDUCED 1	Thr/Ala
chr3_1	91883134	A	C	0	1	0	1	MtrunA17_Chr3g0120731	4° exon	Putative long-chain-fatty-acid-CoA ligase	Stop/Glu
chr4_1	65612430	A	G	0	1	0	0,94	MtrunA17Chr8g0379001	9° exon	Putative alpha/Beta hydrolase, lipase	
chr4_1	65612442	T	A	0	1	0	0,94				
chr4_1	66815261	T	C	0,96	0	1	0	MtrunA17_Chr8g0380461	6° exon	Putative PD2 domain, tetratricopeptide-like helical domain superfamily	
chr8_1	50421996	A	G	0	1	0	1		intergenic region		
chr8_1	80665087	A	T	0	1	0	1	MtrunA17Chr8g0378621	1° intron	Beta-1,3-Glucanase 1	
chr8_1	80665088	A	T	0	1	0	1				

Chromosome	position	Reference allele	Alternative allele	EF5	LF5	EFsyn	LFsyn	Gene name (*M. truncatula* A17 r5.0 genome)	SNP position	Gene function	AA change
chr1_1	35205946	C	G	0	1	0,083	0,93	MtrunA17_Chr1g0171471	3° exon	Putative glycerol-3-phosphate dehydrogenase	
chr2_1	18974900	A	G	0	1	0	1	MtrunA17_Chr2g0294361	1° exon	Phytochrome B	
chr2_1	18974907	T	C	0	1	0	1				
chr2_1	57629906	G	A	1	0	1	0	MtrunA17_Chr2g0314621	17° exon	Trehalose phosphate synthase 4	
chr2_1	68475230	T	C	0	1	0	1	MtrunA17_Chr7g0269581	6° intron	MtSUCS2, sucrose synthase 2	
chr2_1	68555885	A	G	0,98	0	0,98	0	MtrunA17Chr2g0323941	11° exon	Putative non-specific protein-tyrosine kinase RLK-Pelle-LRR-V family	
chr4_1	55246838	A	G	0	1	0	1		intergenic region		
chr4_1	76319199	G	A	1	0	1	0	MtrunA17Chr3g0084091	5° exon	Putative EamA domain-containing protein	Leu/Ser
chr4_1	79577664	G	T	1	0	1	0	no alignement found			
chr7_2	75842177	A	G	0	1	0	1	no alignement found			
chr8_1	10677703	T	C	0	1	0	1	MtrunA17_Chr8g0348881	11° exon	Putative mediator complex, subunit Med 12	Ile/Thr
chr8_1	50421996	A	G	0	1	0	1		intergenic region		

Chromosome	position	Reference allele	Alternative allele	EF6	LF6	EFsyn	LFsyn	Gene name (*M. truncatula* A17 r5.0 genome)	SNP position	Gene function	AA change
chr1_1	35205946	C	G	0	1	0,083	0,93	MtrunA17_Chr1g0171471	3° exon	Putative glycerol-3-phosphate dehydrogenase	
chr1_1	69857191	T	G	0	1	0	1		intergenic region		
chr2_1	17246013	G	A	1	0	1	0	MtrunA17_Chr2g0292681	4° exon	Putative tetratricopeptide repeat protein POLLENLESS 3/ SULPHUR DEFICIENCY INDUCED 1	Thr/Ala
chr3_1	91883134	A	C	0	1	0	1	MtrunA17_Chr3g0120731	4° exon	Putative long-chain-fatty-acid-CoA ligase	Stop/Glu
chr4_1	56175100	C	T	0	1	0	1	MtrunA17_Chr4g0047001	2° exon	Putative transcription factor C2C2-Dof family	
chr4_1	56175124	G	A	0	1	0	1	MtrunA17_Chr4g0047001	2° exon	Putative transcription factor C2C2-Dof family	Val/Ile
chr4_1	74648725	A	C	1	0	1	0		intergenic region		
chr6_1	12497250	C	T	0	0,98	0	1		intergenic region		
chr6_1	81846377	A	G	1	0	0,99	0	no alignement found			

chr7_2	21900732	A	ATTAGAAT	0	1	0	1	MtrunA17_Chr7g0245171	1° intron	Putative protein kinase RLK-Pelle-LRR-III family	
chr7_2	32237110	G	C	0	1	0	0,94	MtrunA17_Chr7g0240901	2° exon	Putative transcription factor interactor and regulator CCHC(Zn) family	

Online Resource 6: List of alfalfa SNPs identified for internode length. Reference allele: allele found in alfalfa genome; Alternative allele: allele found in sequenced plants; SI: short internode lines bulk; LI: long internode lines bulk. Number in columns represent the percentage of alleles differing from reference allele (1=100% difference).

Chromosome	position	Reference allele	Alternative allele	SI 1	SI 2	SI 3	LI 1	LI 2	LI 3
Chr1_1	49970659	G	A	0.96078	0.93878	0.8	0	0	0
Chr2_1	12742137	T	C	1	1	1	0	0	1
Chr2_1	66246619	T	G	1	1	1	0	0	0
Chr2_1	69548749	A	G	0	0	0	1	1	1
Chr3_1	64548426	G	T	0	0	0	1	1	1
Chr3_1	71102283	AT	A	0	0	0.16667	1	0.96	1
Chr4_1	7283617	A	G	1	1	1	0	0	0
Chr4_1	46503437	C	T	0	0	0	0.93878	0.9375	0.94872
Chr5_2	6391112	T	C	0	0	0	1	1	1
Chr5_2	10624251	G	A	0	0	0	1	1	1
Chr6_1	97856076	A	G	0	0	0	1	1	1
Chr7_2	57922206	A	G	0	0	0	0.5	1	1
Chr7_2	76403993	A	G	1	1	1	0	0	0
Chr7_2	76404036	T	C	0	0	0	1	1	1

Online Resource 7 List of the SNPs identified in M. truncatula 1739 homozygous line. Reference allele: allele found in *M. truncatula* A17 r5.0 genome; Alternative allele: allele found in sequenced plants. 0 = homozygous for reference allele; 1 = heterozygous; 2 = homozygous for alternative allele. Difference count: number of alleles found in sequenced plants differing from Jemalong 2HA10-9 (control); LF: late flowering plants

**Chromosom e**	**position**	**Referenc e allele**	**Alternativ e allele**	**Differenc e count**	**1739_1L F**	**1739_2L F**	**1739_3L F**	**1739_4L F**	**1739_5LF**	**1739_6L F**	**1739_7L F**	**1739_8L F**	**1739_9L F**	**Jemalong 2HA10-9**
1	4186225	T	G	4	0	0	0	2	0	0	0	2	0	0
1	4538747	G	A	5	1	2	1	2	1	1	NA	2	1	2
1	6244183	C	T	9	1	0	1	1	1	2	0	2	1	0
1	6472927	G	A	8	1	2	1	2	1	1	0	1	1	2
1	7083980	G	A	2	2	0	2	2	NA	2	2	2	2	2
1	7190715	G	A	11	1	0	1	0	1	0	2	1	1	2
1	9763490	G	A	9	1	0	1	1	1	1	2	1	1	2
1	20217646	TG	T	6	0	0	2	NA	2	2	0	0	0	0
1	20459176	A	T	4	0	0	0	0	2	0	0	2	0	0
1	28891653	G	A	8	1	1	1	1	2	2	1	0	1	2
1	38122402	A	C	2	2	2	2	2	2	1	1	2	2	2
1	38588530	C	T	7	1	1	2	2	1	2	1	1	0	2
1	39305896	T	C	2	0	2	2	2	NA	2	2	2	2	2
1	40028788	CT	C	2	2	NA	2	2	1	1	2	2	2	2
1	40315896	C	T	18	2	2	2	2	2	2	2	2	2	0
1	42540483	C	T	6	1	1	2	2	2	2	1	1	0	2
1	45125012	C	T	7	1	1	1	2	2	2	1	1	0	2
1	46672493	C	T	18	2	2	2	2	2	2	2	2	2	0
1	50703650	A	C	4	0	0	0	0	0	0	0	2	2	0
1	52800029	A	G	2	2	2	2	2	0	2	2	2	2	2
2	1853860	TA	T	2	1	2	2	NA	2	1	2	2	2	2
2	6079380	A	G	2	2	2	2	0	NA	2	2	2	2	2
2	8221005	C	G	7	1	1	1	1	1	2	1	1	2	2
2	8221014	C	T	7	1	1	1	1	1	2	1	1	2	2
2	9702610	A	G	6	1	1	1	2	1	1	1	2	2	2
2	9702618	A	T	6	1	1	1	2	1	1	1	2	2	2
2	9702624	A	G	6	1	1	1	2	1	1	1	2	2	2
2	11645548	T	C	6	2	0	0	2	2	2	2	0	2	2
2	13476159	T	G	3	2	1	2	2	1	2	2	2	1	2
2	13997380	C	T	16	0	0	0	0	0	0	2	0	0	2
2	16820927	G	A	2	2	2	2	2	0	2	2	2	2	2
2	17414494	T	G	3	2	1	1	2	1	2	2	2	2	2

2	17994940	T	C	2	0	0	0	0	0	2	0	0	0	0
2	18946103	C	T	18	0	0	0	0	0	0	0	0	0	2
2	31278900	C	T	8	NA	1	0	2	2	0	1	1	1	0
2	31530957	C	T	18	0	0	0	0	0	0	0	0	0	2
2	39601736	C	T	9	2	2	0	0	1	0	2	2	0	2
2	42905336	C	T	18	0	0	0	0	0	0	0	0	0	2
2	46518696	T	A	7	1	2	1	1	NA	1	1	1	1	2
3	782056	A	C	8	2	0	0	2	0	2	0	NA	2	2
3	3074305	G	A	10	1	1	1	2	1	0	0	2	0	2
3	4012211	A	G	5	1	2	1	1	1	2	2	1	2	2
3	4199596	C	T	18	2	2	2	2	2	2	2	2	2	0
3	8743180	A	G	6	2	0	0	2	0	2	0	0	0	0
3	18420187	T	C	2	0	0	2	0	0	0	0	0	0	0
3	27869078	A	G	2	2	2	2	0	2	2	2	2	2	2
3	30894756	A	G	2	2	2	2	2	2	2	2	1	1	2
3	30894811	T	A	4	2	1	2	1	1	2	2	2	1	2
3	30894830	C	T	2	2	2	2	1	2	2	2	2	1	2
3	35488554	G	A	18	0	0	0	0	0	0	0	0	0	2
3	42791235	G	A	18	2	2	2	2	2	2	2	2	2	0
3	43254344	T	G	3	2	2	1	2	1	2	2	1	2	2
3	45584895	G	A	18	0	0	0	0	0	0	0	0	0	2
3	46041613	A	G	2	2	2	2	0	NA	2	2	2	2	2
3	49997294	G	A	18	0	0	0	0	0	0	0	0	0	2
3	53704904	A	G	2	2	2	2	2	1	2	1	2	2	2
4	352044	A	C	4	0	2	0	2	0	0	0	0	0	0
4	5143228	C	G	3	2	1	1	2	1	2	2	2	2	2
4	5957228	G	A	16	2	2	NA	2	2	2	2	2	2	0
4	9840280	A	G	2	2	2	0	2	2	2	2	2	2	2
4	9840333	A	T	2	2	2	0	2	2	2	2	2	2	2
4	20298935	C	T	16	2	2	2	2	NA	2	2	2	2	0
4	24404523	C	T	13	1	1	0	0	1	1	0	0	1	2
4	30266067	G	A	16	2	2	2	2	NA	2	2	2	2	0
4	30597002	TA	T	2	2	2	2	2	0	2	2	2	2	2
4	36226410	G	A	18	2	2	2	2	2	2	2	2	2	0
4	36724417	T	C	2	2	1	2	2	NA	2	1	2	2	2
4	38092340	C	T	7	1	1	1	2	1	1	2	1	1	2
4	38158556	T	C	2	2	2	2	2	0	2	2	2	2	2
4	40766150	G	A	18	2	2	2	2	2	2	2	2	2	0
4	44896377	G	A	7	1	1	1	2	1	1	2	1	1	2
4	48897561	T	C	2	2	0	2	2	2	2	2	2	2	2
4	49322710	C	T	18	0	0	0	0	0	0	0	0	0	2

4	50350734	T	A	3	2	2	1	1	2	2	1	2	2	2
4	50443296	C	T	18	0	0	0	0	0	0	0	0	0	2
4	51871722	C	T	16	0	0	0	0	NA	0	0	0	0	2
4	64483854	G	A	18	2	2	2	2	2	2	2	2	2	0
5	5313443	G	A	18	2	2	2	2	2	2	2	2	2	0
5	9011449	C	T	18	2	2	2	2	2	2	2	2	2	0
5	10087639	A	C	2	2	2	2	2	2	2	1	2	1	2
5	14568730	C	T	11	2	1	0	1	0	0	1	1	1	2
5	19932544	A	G	2	2	2	2	NA	0	2	2	2	2	2
5	26703532	AC	A	17	2	2	2	2	2	2	1	2	2	0
5	35211794	C	A	2	2	2	1	2	NA	2	1	2	2	2
5	35996706	G	A	18	2	2	2	2	2	2	2	2	2	0
5	39058848	AG	A	2	0	0	0	0	0	0	2	0	0	0
5	39333582	T	G	2	2	2	2	2	0	2	2	2	2	2
5	39463899	C	G	2	2	2	2	0	2	2	2	2	2	2
5	40853460	G	A	16	2	2	2	2	NA	2	2	2	2	0
5	43013859	G	A	16	2	2	2	2	2	2	NA	2	2	0
6	1337680	C	T	18	2	2	2	2	2	2	2	2	2	0
6	4243456	T	G	2	2	2	1	2	1	2	2	2	2	2
6	4818882	G	A	6	1	NA	2	2	1	1	0	1	2	2
6	12252882	A	G	7	1	1	1	1	1	1	NA	1	0	0
6	12252895	G	A	7	1	1	1	1	1	1	NA	1	0	0
6	14509266	A	C	8	0	0	2	2	0	2	2	2	0	2
6	15298165	T	A	2	0	0	0	0	NA	2	0	0	0	0
6	15298167	T	G	2	0	0	0	0	NA	2	0	0	0	0
6	14966040	G	T	2	NA	0	0	0	0	0	2	0	0	0
6	14966037	C	T	2	NA	0	0	0	0	0	2	0	0	0
6	14964394	A	G	12	0	0	0	0	NA	0	0	2	2	2
6	15931534	G	A	10	0	1	0	0	1	2	1	2	1	2
6	16188084	G	A	2	2	2	1	2	NA	2	2	1	2	2
6	20451475	T	G	9	1	1	1	1	1	1	1	1	1	2
6	31562938	C	T	9	1	1	1	0	2	1	2	0	1	2
6	32112337	A	G	2	2	2	2	1	1	2	2	2	2	2
6	35883507	A	C	3	1	2	2	2	NA	2	2	1	1	2
6	36558188	G	A	10	2	2	0	0	2	0	0	2	0	2
6	39575940	T	C	12	2	2	0	0	0	2	0	0	0	2
6	41555486	C	T	18	2	2	2	2	2	2	2	2	2	0
6	42645969	T	C	2	2	NA	1	2	2	1	2	2	2	2
7	9263617	C	T	16	0	0	0	0	0	0	NA	0	0	2
7	10413123	G	A	5	1	2	2	2	1	2	1	1	1	2
7	11942546	C	T	18	0	0	0	0	0	0	0	0	0	2

7	27001168	G	A	2	2	2	2	0	NA	2	2	2	2	2
7	29545932	A	G	2	2	0	2	2	NA	2	2	2	2	2
7	37108355	C	T	18	2	2	2	2	2	2	2	2	2	0
7	38087322	T	G	10	0	2	2	2	0	2	2	NA	0	0
7	45012438	C	T	18	0	0	0	0	0	0	0	0	0	2
7	45065601	G	A	18	0	0	0	0	0	0	0	0	0	2
7	48259765	C	T	18	2	2	2	2	2	2	2	2	2	0
7	48472569	C	T	18	2	2	2	2	2	2	2	2	2	0
7	49340664	C	T	18	2	2	2	2	2	2	2	2	2	0
7	49828684	A	G	2	0	0	0	0	0	0	NA	2	0	0
7	51362536	G	A	18	0	0	0	0	0	0	0	0	0	2
7	55044464	A	G	2	1	2	2	2	NA	2	1	2	2	2
8	1679834	G	A	18	2	2	2	2	2	2	2	2	2	0
8	7132036	C	G	16	0	0	0	0	0	0	0	0	2	2
8	8939469	G	A	18	0	0	0	0	0	0	0	0	0	2
8	11267494	A	G	2	2	NA	0	2	2	2	2	2	2	2
8	13478170	C	T	18	0	0	0	0	0	0	0	0	0	2
8	15278931	G	A	16	0	0	0	0	0	0	NA	0	0	2
8	18905390	G	A	4	2	2	1	2	2	1	1	1	2	2
8	27360493	A	T	3	1	2	2	1	NA	1	2	2	2	2
8	27848259	T	C	5	1	2	0	1	1	2	2	2	2	2
8	27848286	C	T	7	1	1	1	1	2	2	1	1	1	2
8	31519091	TA	T	2	0	0	2	0	0	0	NA	0	0	0
8	34140083	G	A	18	2	2	2	2	2	2	2	2	2	0
8	49691016	C	T	11	1	1	1	0	1	1	0	1	1	2

Online Resource 8 List of the SNPs identified in M. truncatula 2345 segregating line. Reference allele: allele found in *M. truncatula* A17 r5.0 genome; Alternative allele: allele found in sequenced plants. 0 = homozygous for reference allele; 1 = heterozygous; 2 = homozygous for alternative allele. Difference count: number of alleles found in sequenced plants differing from 2345_LF bulk; LF: late flowering

Chromosome	position	Reference allele	Alternative allele	Difference count	2345_1	2345_2	2345_3	2345_4	2345_5	2345_6	2345_7	2345_LF
1	4186225	T	G	10	2	0	0	0	0	0	NA	2
1	5107881	T	C	2	2	NA	1	2	2	2	1	2
1	5160129	C	T	7	1	1	2	2	0	0	1	2
1	20217646	TG	T	8	2	2	2	0	0	2	0	0
1	29467573	T	C	4	2	NA	0	0	2	0	0	0
1	27935143	T	C	2	1	1	2	2	NA	2	2	2
1	28995248	A	G	2	2	1	2	2	2	1	2	2
1	34450164	C	T	8	1	0	0	1	1	1	2	2
1	34747727	TA	T	2	2	2	2	1	2	1	2	2
1	39470281	C	T	10	1	0	0	1	1	0	1	2
1	40315896	C	T	10	1	2	2	1	1	2	1	0
1	41936336	TAC	T	2	2	2	0	2	2	2	2	2
1	41936340	A	T	2	2	2	2	2	2	0	2	2
1	45201433	C	T	9	1	0	1	1	1	0	1	2
1	45703412	C	T	3	2	2	1	1	2	2	1	2
1	46406388	C	T	10	1	0	1	1	0	0	1	2
1	46672493	C	T	10	1	2	1	1	2	2	1	0
1	46735362	AT	A	2	0	0	2	0	0	0	0	0
1	47458232	T	C	10	2	2	0	2	2	2	NA	0
1	52927897	A	T	2	0	0	2	0	0	0	0	0
1	52927904	A	T	2	0	0	2	0	0	0	0	0
1	55286149	C	T	2	1	2	2	2	2	1	2	2
1	56557638	G	A	9	2	0	1	1	0	0	1	2
2	1793509	G	A	11	2	1	2	2	1	1	2	0
2	8057802	T	C	2	2	2	2	2	2	0	2	2
2	8221005	C	G	2	2	1	2	2	2	1	2	2
2	8221014	C	T	2	2	1	2	2	2	1	2	2
2	9869499	T	C	2	0	0	2	0	0	0	0	0
2	11510686	A	G	2	2	2	2	2	2	1	1	2
2	11645548	T	C	10	2	0	2	2	0	2	2	0
2	17994940	T	C	2	0	0	2	0	0	0	0	0
2	18944497	T	C	2	2	2	NA	2	2	0	2	2
2	21262208	G	T	3	1	1	2	1	2	2	NA	2

2	24048087	A	T	3	2	1	1	1	2	2	NA	2
2	46107791	G	A	2	0	0	0	0	0	2	NA	0
2	48144794	A	G	2	1	1	2	2	2	2	2	2
3	782056	A	C	8	0	0	2	2	0	0	NA	2
3	4012155	T	C	8	1	0	1	1	0	1	2	2
3	14415960	G	A	2	2	1	2	1	2	2	NA	2
3	30894756	A	G	3	2	1	2	2	1	2	1	2
3	30894811	T	A	3	2	2	1	2	1	2	1	2
3	30894830	C	T	2	2	2	2	2	1	2	1	2
3	43254344	T	G	2	2	1	2	2	1	2	2	2
3	53521759	CA	C	2	2	2	2	1	2	1	NA	2
4	352044	A	C	2	0	0	0	0	2	0	0	0
4	1506627	G	A	2	1	2	1	2	2	2	2	2
4	3345239	C	T	6	1	1	1	1	1	1	NA	2
4	3345241	T	C	6	1	1	1	1	1	1	NA	2
4	12803065	G	GT	2	0	0	0	0	0	2	0	0
4	12591761	C	CT	2	0	0	0	0	0	0	2	0
4	36543394	GT	G	4	2	2	NA	0	0	0	0	0
4	37047121	CT	C	4	0	2	2	0	0	0	NA	0
4	43582125	G	A	6	1	0	1	1	2	1	0	0
4	57754456	A	G	2	2	NA	2	2	1	1	2	2
4	59122601	A	T	8	2	0	2	2	0	0	0	2
4	62814389	T	G	2	2	1	2	2	2	1	NA	2
4	63149264	A	G	2	2	2	2	2	0	2	2	2
5	5313443	G	A	8	1	0	0	2	1	2	0	2
5	6211596	G	T	2	0	0	0	0	2	0	0	0
5	6211642	A	G	2	0	0	0	0	2	0	0	0
5	6211650	T	C	2	0	0	0	0	2	0	0	0
5	6458942	A	G	2	2	2	2	1	2	1	2	2
5	8850065	C	T	2	0	0	0	0	2	0	0	0
5	9011449	C	T	8	1	0	0	2	1	2	0	2
5	26703532	AC	A	6	2	2	0	2	2	0	0	2
5	29119518	A	G	12	0	2	0	0	0	0	0	2
5	29119525	T	C	12	0	2	0	0	0	0	0	2
5	33646424	TA	T	2	2	2	2	1	2	1	2	2
5	34431150	A	G	7	1	2	1	1	1	1	NA	0
5	37421050	A	G	2	2	2	2	2	2	2	0	2
5	39463899	C	G	2	2	2	0	2	2	2	2	2
6	7434870	T	C	2	2	2	2	2	2	2	0	2
6	10937455	C	T	2	2	2	2	1	2	2	1	2
6	14509266	A	C	12	0	0	0	0	0	2	0	2

6	15365234	T	C	2	2	2	2	1	2	2	1	2
6	14964617	A	T	2	0	0	0	2	0	0	0	0
6	14964394	A	G	6	0	0	2	2	0	0	2	0
6	16438350	A	AT	2	0	0	0	0	0	2	0	0
6	39575940	T	C	10	0	0	2	2	0	0	0	2
7	9688596	A	T	2	0	0	0	0	2	0	0	0
7	9688612	A	C	2	0	0	0	0	2	0	0	0
7	10413004	A	G	3	1	2	1	2	2	1	2	2
7	10413096	G	A	4	1	1	1	2	2	1	2	2
7	10413123	G	A	2	1	2	1	2	2	2	2	2
7	38087322	T	G	6	0	0	2	2	2	0	2	2
7	43030775	G	C	2	1	2	2	2	1	2	2	2
7	47134849	ATC	A	2	2	2	2	2	1	2	1	2
8	7132036	C	G	2	2	0	0	0	0	0	NA	0
8	18905390	G	A	2	1	2	2	1	2	2	NA	2
8	27360493	A	T	3	2	1	2	2	1	2	1	2
8	27848259	T	C	5	1	2	2	1	1	1	1	2
8	27848286	C	T	3	1	2	1	1	2	2	2	2
8	30278067	C	T	9	1	0	0	0	2	2	0	2
8	35565542	T	C	2	1	2	2	2	1	NA	2	2
8	37915579	G	A	10	2	2	NA	2	2	2	0	0
8	37915580	C	T	10	2	2	NA	2	2	2	0	0

Online Resource 9 GO terms (level 3) of the genes carrying SNP in analysed lines: a) 91 genes found in LF vs EF lines of alfalfa (grey) and LF vs EF plants of *M. truncatula* (withe). b) 11 genes found in SI vs LI alfalfa synthetics. NA: not annotated

a)

Gene (*M. truncatula* A17)	Biological Process	Cellular Component	Molecular Function	ShinyGO significantly enriched terms	Line with SNP
MtrunA17Chr1g0155351	NA	GO 0005737: cytoplasm<br>GO 0043226: organelle	GO 00055015: protein binding	GO 00055015: protein binding	Mt1739
MtrunA17Chr1g0158991	GO 0051234: establishment of localization	GO 0005737: cytoplasm<br>GO 0043226: organelle<br>GO 0016020: membrane<br>GO 0012505: endomembrane system	GO 0022857: transmembrane transporter activity		Mt1739
MtrunA17Chr1g0171471	NA	NA	NA		EF4-LF4, EF5-LF5, EF6-LF6
MtrunA17Chr1g0176251	NA	GO 0016020: membrane	GO 0016740: transferase activity		EF1-LF1
MtrunA17Chr1g0183781	GO 0008152: metabolic process	GO 0005737: cytoplasm<br>GO 0043226: organelle	GO 0140096: catalitic activity acting on a protein<br>GO 0016740: transferase activity<br>GO 0016874: ligase activity<br>GO 00055015: protein binding	GO 00055015: protein binding<br>GO 0003677: DNA binding<br>GO 0016874: ligase activity	Mt2345
MtrunA17Chr1g0183871	GO 0071840: cellular component organization or biogenesis<br>GO 0007049: cell cycle	GO 0043226: organelle	GO 00055015: protein binding<br>GO 0036094: small molecul binding<br>GO 0003676: nucleic acid binding	GO 00055015: protein binding<br>GO 0005524 ATP binding	EF2-LF2
MtrunA17Chr1g0190061	NA	NA	NA		EF2-LF2
MtrunA17Chr1g0190201	NA	NA	GO 00055015: protein binding	GO 00055015: protein binding	Mt2345
MtrunA17Chr1g0191161	NA	NA	GO 0016740: transferase activity<br>GO 0140096: catalityc activity acting on a protein		EF1-LF1, EF2-LF2
MtrunA17Chr1g0191211	NA	NA	NA		Mt2345
MtrunA17Chr1g0198131	GO 0008152: metabolic process	NA	GO 0140096: catalitic activity acting on a protein<br>GO 0016787: Hydrolase activity		Mt2345
MtrunA17Chr1g0198461	NA	NA	GO 0003676 nucleic: acid binding		EF4-LF4
MtrunA17Chr1g0199691	GO 0008152: metabolic process	GO 0005737: cytoplasm<br>GO 0043226: organelle<br>GO 0009532: plastid stroma	GO 0016858: isomerase activity<br>GO 0016740: transferase activity<br>GO 0036094: small molecul binding	GO 0004615: phosphomannomutase activity	Mt2345

MtrunA17Chr1g0200031	GO 0009605: response to external stimulus<br>GO 0009607: response to biotic stimulus<br>GO 0006950: response to stress	GO 0005737: cytoplasm<br>GO 0043226: organelle<br>GO 0016020: membrane<br>GO 0012505: endomembrane system	NA		Mt2345
MtrunA17Chr1g0213771	GO 0008152: metabolic process	GO 0016020: membrane	GO 0016787: hydrolase activity		Mt2345
MtrunA17Chr2g0279331	NA	NA	NA		Mt2345
MtrunA17Chr2g0292681	NA	NA	GO 00055015: protein binding<br>GO 0016740: transferase activity	GO 00055015: protein binding	EF2-LF2, EF4-LF4, EF6-LF6
MtrunA17Chr2g0294361	GO 0050789: regulation of biological process	GO 0043226: organelle	GO 0140096: catalytic activity acting on a protein<br>GO 0016740: transferase activity<br>GO 0038023: signaling receptor activity		EF5-LF5
MtrunA17Chr2g0295561	GO 0071840: cellular component organization or biogenesis	GO 0016020: membrane<br>GO 0043226: organelle<br>GO 0030312: external encapsulating structure	GO 0016787: hydrolase activity<br>GO 0003682: chromatin binding	GO 0004857: enzyme inhibitor activity	Mt1739
MtrunA17Chr2g0301251	GO 005078: regulation of biological process	GO 0043226: organelle	GO 0003676: nucleic acid binding<br>GO 00055015: protein binding	GO 00055015: protein binding<br>GO 0003677: DNA binding	Mt1739
MtrunA17Chr2g0308681	GO 0008152: metabolic process	GO 0005737: cytoplasm<br>GO 0043226: organelle<br>GO 0012505: endomembrane system	GO 0140096: catalitic activity acting on a protein<br>GO 0016740: transferase activity<br>GO 00055015: protein binding		Mt1739
MtrunA17Chr2g0314621	GO 0008152: metabolic process	GO 0005737: cytoplasm	GO 0016740: transferase activity		EF5-LF5
MtrunA17Chr2g0323941	GO 0051234: establishment of localization<br>GO 0033036: macromolecul localization	GO 0005737: cytoplasm<br>GO 0043226: organelle<br>GO 0012505: endomembrane system	GO 0140096: catalytic activity acting on a protein<br>GO 0016740: transferase activity<br>GO 0038023: signaling receptor activity	GO 0005524 ATP binding	EF2-LF2, EF5-LF5
MtrunA17Chr3R0023360	NA	NA	NA		Mt1739
MtrunA17Chr3g0084091	GO 0051234: establishment of localization	GO 0016020: membrane	GO 0022857: transmembrane transporter activity		EF1-LF1, EF2-LF2, EF5-LF5
MtrunA17Chr3g0113821	NA	NA	NA		Mt1739
MtrunA17Chr3g0120731	GO 0008152: metabolic process	GO 0005737: cytoplasm<br>GO 0043226: organelle<br>GO 0016020: membrane<br>GO 0012505: endomembrane system	GO 0016874: ligase activity	GO 0016874: ligase activity	EF1-LF1, EF3-LF3, EF4-LF4, EF6-LF6

MtrunA17Chr3g0123691	GO 005078: regulation of biological process	GO 0043226: organelle	GO 0003676 nucleic: acid binding		Mt1739
MtrunA17Chr3g0127571	GO 0008152: metabolic process<br>GO 0050789: regulation of biological process	GO 0005737: cytoplasm<br>GO 0043226: organelle	GO 0140096: catalytic activity acting on a protein	GO 0005524 ATP binding	Mt1739
MtrunA17Chr3g0133511	NA	GO 0005615: extracellular space	GO 0016787: Hydrolase activity<br>GO 0030234: enzyme regulator activity		Mt1739
MtrunA17Chr3g0144041	GO 0051234: establishment of localization	NA	GO 0036094: small molecul binding<br>GO 0005515: protein binding	GO 00055015: protein binding	EF1-LF1
MtrunA17Chr3g0144181	GO 0051234: establishment of localization<br>GO 0033036: macromolecul localization<br>GO 0006950: response to stress<br>GO 0009607: response to biotic stimulus	GO 0005737: cytoplasm<br>GO 0043226: organelle<br>GO 0016020: membrane<br>GO 0012505: endomembrane system	GO 0016740: transferase activity		EF1-LF1
MtrunA17Chr4g0007641	GO 0071840: cellular component organization or biogenesis	GO 0043226: organelle	GO 0016740: transferase activity<br>GO 0003682: chromatin binding	GO 0003677: DNA binding	Mt1739
MtrunA17Chr4g0021701	GO 0008152: metabolic process	GO 0016020: membrane	GO 0016787: hydrolase activity	GO 0016798: hydrolase activity-acting on glycosyl bonds	Mt1739
MtrunA17Chr4g0023841	GO 0051234: establishment of localization<br>GO 0051641: cellular localization<br>GO 0033036: macromolecul localization<br>GO 0008152: metabolic process<br>GO 0006457: protein folding	GO 0005737: cytoplasm<br>GO 0043226: organelle	GO 0036094: small molecul binding<br>GO 0005515: protein binding	GO 00055015: protein binding	Mt1739
MtrunA17Chr4g0026411	NA	NA	NA		EF1-LF1
MtrunA17Chr4g0029431	GO 0071840: cellular component organization or biogenesis	GO 0016020: membrane	NA		Mt1739
MtrunA17Chr4g0036011	GO 0008152: metabolic process<br>GO 0006950: response to stress		GO 0016829: lyase activity		EF1-LF1
MtrunA17Chr4g0037691	GO 0051716: cellular response to stimulus<br>GO 0006950: response to stress<br>GO 0008152: metabolic process	NA	GO 0003676: nucleic acid binding<br>GO 0140640: catalytic activity acting on nucleic acid	GO 0005524 ATP binding<br>GO 0003677: DNA binding	Mt1739

MtrunA17Chr4g0040511	GO 0050789: regulation of biological process	GO 0043226: organelle	GO 0003676 nucleic: acid binding	GO 0003677: DNA binding	EF2-LF2
MtrunA17Chr4g0043781	GO 0051716: cellular response to stimulus<br>GO 0006950: response to stress	GO 0005737: cytoplasm<br>GO 0043226: organelle	GO 0003824: catalytic activity	GO 0005524 ATP binding	Mt1739
MtrunA17Chr4g0047001	GO 0050789: regulation of biological process<br>GO 0008152: metabolic process	GO 0043226: organelle	GO 0003676 nucleic: acid binding	GO 0003677: DNA binding	EF6-LF6
MtrunA17Chr4g0052341	NA	NA	GO 0036094: small molecul binding<br>GO 0140640: catalitic activity acting on nucleic acid<br>GO0016787: hydrolase activity	GO 0005524 ATP binding<br>GO 0070615: nucleosome-dependent ATPase activity	EF2-LF2
MtrunA17Chr4g0055761	GO 005078: regulation of biological process	NA	GO 0003676: nucleic acid binding<br>GO 0140640: catalytic activity acting on nucleic acid	GO 0003677: DNA binding	Mt1739
MtrunA17Chr4g0057141	GO 005078: regulation of biological process	GO 0043226: organelle<br>GO 1990904: ribonucleoprotein complex	GO 0140640: catalytic activity acting on nucleic acid<br>GO 0005515: protein binding	GO 00055015: protein binding	Mt1739
MtrunA17Chr4g0059061	NA	NA	NA		Mt1739
MtrunA17Chr4g0075951	GO 0051234: establishment of localization	GO 0005737: cytoplasm	GO 0036094: small molecul binding		EF1-LF1,EF3-LF3
MtrunA17Chr4g0076731	GO 005078: regulation of biological process	NA	GO 0003676 nucleic: acid binding	GO 0003677: DNA binding	Mt1739
MtrunA17Chr4g0077061	NA	GO 0016020: membrane	GO 0003676: nucleic acid binding<br>GO 0140640: catalitic activity acting on nucleic acid<br>GO0016787: hydrolase activity	GO 0005524 ATP binding	EF1-LF1
MtrunA17Chr5g0401061	GO 0071840: cellular component organization or biogenesis	GO 0043226: organelle	GO 0003676 nucleic: acid binding		Mt1739
MtrunA17Chr5g0405891	GO 0008152: metabolic process	GO 0005737: cytoplasm<br>GO 0043226: organelle	GO 0016740: transferase activity<br>GO 0005515: protein binding<br>GO 0016874: ligase activity	GO 00055015: protein binding<br>GO 0016874: ligase activity	Mt1739
MtrunA17Chr5g0413331	NA	GO 0016020: membrane	GO 0016740: transferase activity<br>GO 0016829: lyase activity<br>GO 0016874: ligase activity	GO 0005524 ATP binding<br>GO 0003878: ATP citrate synthase activity<br>GO 0016874: ligase activity	Mt1739
MtrunA17Chr5g0424671	NA	NA	NA		Mt1739

MtrunA17Chr5g0427261	NA	NA	NA		EF3-LF3
MtrunA17Chr5g0427271	NA	NA	NA		Mt2345
MtrunA17Chr5g0434281	GO 0008152: metabolic process	GO 0005737: cytoplasm<br>GO 1902494: catalytic complex	GO 0140640: catalytic activity acting on a nucleic acid<br>GO 0036094: small molecul binding<br>GO 0003676: nucleic acid binding	GO 0005524 ATP binding	Mt2345
MtrunA17Chr5g0436081	GO 0008152: metabolic process<br>GO 0050789: regulation of biological process	GO 0043226: organelle<br>GO 0005828: kinetochore microtubule	GO 0036094: small molecul binding<br>GO 0005515: protein binding	GO 00055015: protein binding<br>GO 0005524 ATP binding	Mt1739
MtrunA17Chr5g0442821	GO 0008152: metabolic process	GO 0005737: cytoplasm<br>GO 1902494: catalytic complex	GO 00055015: protein binding		Mt1739
MtrunA17Chr5g0446431	GO 0008152: metabolic process	GO 0005737: cytoplasm<br>GO 0016020: membrane	GO 0016740: transferase activity		Mt1739
MtrunA17Chr6g0450571	GO 0008152: metabolic process	NA	GO 0140096: catalytic activity acting on a protein<br>GO 0016740: transferase activity	GO 0005524 ATP binding	EF2-LF2
MtrunA17Chr6g0450621	NA	NA	NA		Mt1739
MtrunA17Chr6g0465291	GO 0008152: metabolic process	GO 0016020: membrane	GO 00055015: protein binding<br>GO 0016740: transferase activity<br>GO 0140096: catalitic activity acting on a protein<br>GO 0016787: Hydrolase activity	GO 00055015: protein binding<br>GO 0016798: hydrolase activity-acting on glycosyl bonds	Mt2345
MtrunA17Chr6g0470341	NA	NA	NA		Mt1739
MtrunA17Chr6g0487261	GO 0008152: metabolic process	GO 0016020: membrane	GO 0016787: hydrolase activity		Mt1739
MtrunA17Chr7g0224051	GO 0050789: regulation of biological process<br>GO 0071840: cellular component organization or biogenesis	NA	GO 0036094: small molecul binding<br>GO 0140640: catalitic activity acting on nucleic acid<br>GO0016787: hydrolase activity	GO 0005524 ATP binding<br>GO 0070615: nucleosome-dependent ATPase activity	Mt1739
MtrunA17Chr7g0226791	GO 0008152: metabolic process	NA	GO 0140096: catalitic activity acting on a protein<br>GO 0016740: transferase activity		Mt1739
MtrunA17Chr7g0237821	GO 0008152: metabolic process	GO 0005737: cytoplasm<br>GO 0043226: organelle<br>GO 0016020: membrane<br>GO 0012505: endomembrane system	GO 0140096: catalytic activity acting on a protein<br>GO 0016740: transferase activity<br>GO 005515: protein binding	GO 00055015: protein binding<br>GO 0005524 ATP binding	EF2-LF2
MtrunA17Chr7g0240901	GO 0008152: metabolic process	GO 0043226: organelle	GO 0036094: small molecul binding<br>GO 0140640: catalitic activity acting on nucleic acid		EF1-LF1, EF6-LF6

MtrunA17Chr7g0245171	GO 0008152: metabolic process	GO 0005737: cytoplasm<br>GO 0043226: organelle<br>GO 0016020: membrane<br>GO 0012505: endomembrane system	GO 0140096: catalytic activity acting on a protein<br>GO 0016740: transferase activity	GO 0005524 ATP binding	EF6-LF6
MtrunA17Chr7g0249241	GO 0008152: metabolic process	GO 0043226: organelle<br>GO 0016020: membrane	GO 0003676: nucleic acid binding<br>GO 00055015: protein binding<br>GO 0036094: small molecul binding	GO 00055015: protein binding	Mt1739
MtrunA17Chr7g0252141	GO 0050789: regulation of biological process<br>GO 0008152: metabolic process	GO 0005737: cytoplasm	GO 0003676 nucleic: acid binding		EF2-LF2
MtrunA17Chr7g0260161	GO 0008152: metabolic process	GO 0016020: membrane	GO 00055015: protein binding<br>GO 0016740: transferase activity	GO 00055015: protein binding	Mt1739
MtrunA17Chr7g0264521	GO 0051234: establishment of localization<br>GO 0033036: macromolecul localization	GO 0005737: cytoplasm<br>GO 0043226: organelle<br>GO 0016020: membrane<br>GO 0012505: endomembrane system	GO 0036094: small molecul binding<br>GO0016787: hydrolase activity	GO 0005524 ATP binding	EF3-LF3
MtrunA17Chr7g0264841	NA	GO 0016020: membrane	GO 0003676: nucleic acid binding	GO 0003677: DNA binding	Mt1739
MtrunA17Chr7g0265071	NA	NA	NA		Mt1739
MtrunA17Chr7g0269361	GO 0065009: regulation of molecular function	GO 0016020: membrane	GO 0016787: hydrolase activity<br>GO 0030234: enzyme regulator activity	GO 0004857: enzyme inhibitor activity	Mt1739
MtrunA17Chr7g0269581	GO 0008152: metabolic process	NA	GO 0016740: transferase activity	-	EF5-LF5
MtrunA17Chr7R0277390	NA	NA	NA		Mt1739
MtrunA17Chr8g0336451	GO 0008152: metabolic process<br>GO 0050789: regulation of biological process	GO 0005737: cytoplasm	GO 0003676: nucleic acid binding		Mt1739
MtrunA17Chr8g0343811	GO 0008152: metabolic process<br>GO 0050789: regulation of biological process	GO 0043226: organelle<br>GO 0016020: membrane	GO 0003676: nucleic acid binding<br>GO 00055015: protein binding	GO 00055015: protein binding<br>GO 0003677: DNA binding	Mt1739
MtrunA17Chr8g0345971	GO 0008152: metabolic process	GO 0016020: membrane	GO 0140096: catalytic activity acting on a protein		Mt1739
MtrunA17Chr8g0346601	NA	NA	NA		EF2-LF2
MtrunA17Chr8g0348881	NA	NA	NA		EF5-LF5
MtrunA17Chr8g0351341	NA	NA	NA		Mt1739
MtrunA17Chr8g0353481	GO 0008152: metabolic process	GO 0005737: cytoplasm<br>GO 0043226: organelle	GO 0044877: protein-containing complex binding		Mt1739
MtrunA17_Chr8g0376321	GO 0008152: metabolic process<br>GO 0051234: establishment of localization	GO 0043226: organelle<br>GO 0016020: membrane GO 0005737: cytoplasm	GO 0022857: transmembrane transporter activity<br>GO 0016787: Hydrolase activity		Mt2345

MtrunA17Chr8g0378621	GO 0008152: metabolic process	GO 0005737: cytoplasm<br>GO 0043226: organelle<br>GO 0016020: membrane<br>GO 0012505: endomembrane system	GO 0016787: hydrolase activity		EF4-LF4
MtrunA17Chr8g0379001	GO 0008152: metabolic process	NA	GO 0016787: hydrolase activity		EF4-LF4
MtrunA17Chr8g0380461	NA	NA	GO 00055015: protein binding	GO 00055015: protein binding	EF4-LF4
MtrunA17Chr8g0388131	GO 0008152: metabolic process	NA	GO 0016740: transferase activity<br>GO 0036094: small molecul binding	GO 0005524 ATP binding	EF3-LF3
MtrunA17Chr8g0393341	GO 0008152: metabolic process	GO 0016020: membrane	GO 0016787: hydrolase activity<br>GO 0140640: catalytic activity acting on a nucleic acid<br>GO 0016740: transferase activity<br>GO 0005515: protein binding	GO 00055015: protein binding<br>GO 0005524 ATP binding	Mt1739

b)

**Gene (*M. truncatula* A17)**	**Biological process**	**Cellular component**	**Molecular function**
MtrunA17Chr1g0181651	GO 0008152: metabolic process	NA	GO 0003676: nucleic acid binding<br>GO 0016740: transferase activity<br>GO 0140096: catalytic activity acting on a protein
MtrunA17Chr2g0288441	GO 0008152: metabolic process	GO 0043226: organelle<br>GO 0016020: membrane	GO 0022857: transmembrane transporter activity<br>GO 0016874: ligase activity
MtrunA17Chr2g0321101	GO 0008152: metabolic process	GO 0005737: cytoplasm<br>GO 0043226: organelle<br>GO 0016020: membrane<br>GO 0071944: cell periphery	GO 0140096: catalytic activity acting on a protein<br>GO 0016740: transferase activity<br>GO 0036094: small molecul binding
MtrunA17Chr3g0111011	GO 0050789: regulation of biological process<br>GO 0071840: cellular component organization or biogenesis<br>GO 0008152: metabolic process<br>GO 0051716: cellular response to stimulus	GO 0043226: organelle<br>GO 0032993: protein-DNA complex	GO 0003676: nucleic acid binding<br>GO 00055015: protein binding<br>GO 0016740: transferase activity

MtrunA17Chr4g0036011	GO 0008152: metabolic process<br>GO 0006950: response to stress<br>GO 0009628: response to abiotic stimulus	NA	GO 0016829: lyase activity
MtrunA17Chr4g0035881	GO 0008152: metabolic process	NA	GO 0016787: hydrolase activity<br>GO 0140640: catalytic activity acting on a nucleic acid<br>GO 0016740: transferase activity
MtrunA17Chr3g0128551	GO 0008152: metabolic process	GO 0016020: membrane	GO 0140096: catalytic activity acting on a protein<br>GO 0016740: transferase activity<br>GO 0038023: signaling receptor activity<br>GO 0036094: small molecul binding
MtrunA17Chr5g0401551	GO 0008152: metabolic process<br>GO 0050789: regulation of biological process	NA	GO 00055015 protein binding
MtrunA17Chr1g0148461	GO 0008152: metabolic process<br>GO 0050789: regulation of biological process<br>GO 0051716: cellular response to stimulus<br>GO0007154 cell comunication	GO 0016020: membrane<br>GO 0071944: cell periphery	GO 0140096: catalytic activity acting on a protein<br>GO 0016740: transferase activity<br>GO 0036094: small molecul binding<br>GO 0030246 carbohydrate binding
MtrunA17Chr7g0244271	GO 0051234: establishment of localization<br>GO 0051641: cellular localization<br>GO 0033036: macromolecul localization<br>GO 0071840: cellular component organization or biogenesis	GO 0005737: cytoplasm<br>GO 0043226: organelle<br>GO 0016020: membrane<br>GO 0098796: membrane protein complex	GO 0030674 protein-macromolecul adaptor activity<br>GO 00055015 protein binding
MtrunA17Chr7g0262051	NA	GO 0005737: cytoplasm<br>GO 1990904 ribonucleoprotein complex	GO 0003676: nucleic acid binding

Online Resource 10: Gene ontology (GO) classification analysis of the 11 genes carrying SNP in LI vs SI alfalfa synthetics. a) Level 3 GO terms for Biological Process (BP); b) level 3 GO terms for Cellular Component (CC); c) level 3 GO terms for Molecular Function (MF). The numbers in the segments represent the genes belonging to each GO terms; each gene can be present in more than one GO term.

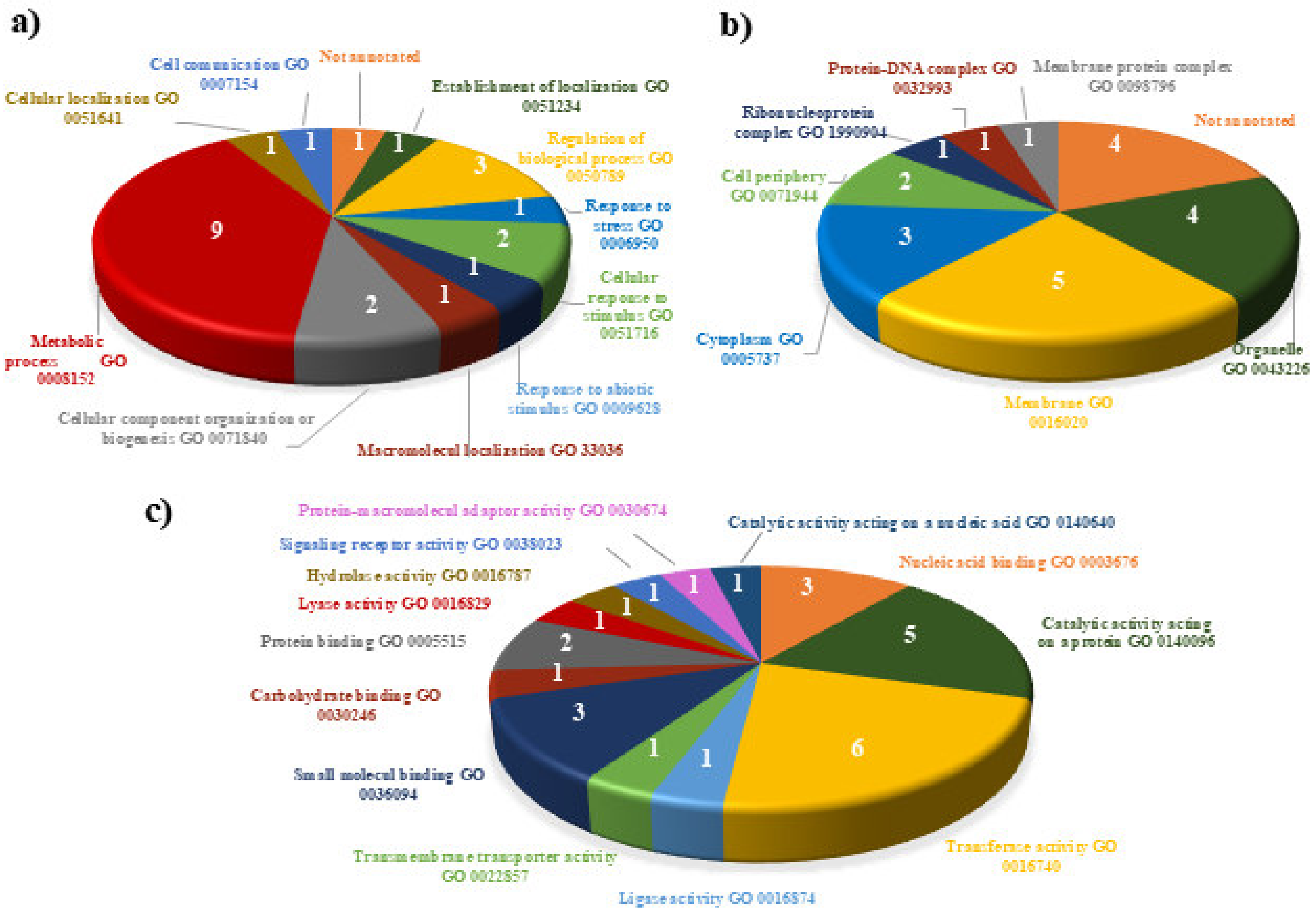

Online Resource 11 Protein structural disruption analysis. ΔΔG values for the three metrics mCSM, SDM and DUET describing the impact of the mutation on the protein structural stability. Amino acid in WT: amino acid present in EF or LI for M. sativa, in Jemalong genotype 2HA10-9 for 1739 line and in wild type plants for 2345 line; amino acid in mutant: alternative amino acid

Line	Protein	Amino acid mutation position	Amino acid in WT	Amino acid in mutant	mCSM kcal/mol	SDM kcal/mol	DUET kcal/mol
**Flowering time**							
M. sativa EF/LF	Putative tetratricopeptide repeat protein POLLENLESS 3 MtrunA17Chr2g0292681	434	T	A	-0.135	0.160	0.133
M. sativa EF/LF	Putative long chain fatty acid CoA ligase MtrunA17Chr3g0120731	337	STOP	E	----	----	---
M. sativa EF/LF	Putative EamA domain-containing protein MtrunA17Chr3g0084091	273	L	S	-0.158	-1.41	-0.065
M. sativa EF/LF	Putative interactor CCHC(Zn) MtrunA17Chr7g0240901	76	Q	E	-0.587	0.540	-0.129
M. sativa EF/LF	Putative WEB family protein MtrunA17Chr4g0075951	191	T	A	-0.529	1.98	0.174
M. truncatula 1739	Phosphate transporter *MtPt4* (AY116211) MtrunA17Chr1g0158991	437	V	A	-1.92	-0.69	-1.893
M. truncatula 1739	Putative protein MtrunA17Chr1g0200031	178	A	T	-0.739	-0.760	-0.540
M. truncatula 1739	MtrunA17Chr2g0301251	238	I	V	-0.555	-0.030	-0.251
M. truncatula 1739	Putative transcription factor C2H2 family MtrunA17Chr2g0308681	234	E	G	-0.022	0.670	0.355
M. truncatula 1739	MtrunA17Chr3g0113821	83	N	D	0.096	-0.13	0.389
M. truncatula 1739	Putative Homobox-WOX transcription factor MtrunA17Chr3g0123691	281	A	T	-0.516	-2	-0.548
M. truncatula 1739	MtrunA17Chr3g0133511	215	N	S			
M. truncatula 1739	MtrunA17Chr4g0007641	3	R	C	-0.093	-0.200	-0.090
M. truncatula 1739	MtrunA17Chr4g0021701	176	R	K	-0.373	-0.090	-0.110

M. truncatula 1739	Putative fasciclin-like arabinogalactan protein MtrunA17Chr4g0029431	184	G	E	-0.536	-2.810	-0.753
M. truncatula 1739	MtrunA17Chr4g0055761	154	E	G	-0.583	3.870	0.382
M. truncatula 1739	Putative transcription factor WD40 family MtrunA17Chr4g0057141	62	N	D	0.161	0.150	0.523
M. truncatula 1739	Putative NAM family transcription factor MtrunA17Chr4g0076731	330	G	S	-0.917	-1.56	-0.884
M. truncatula 1739	Putative transcription factor WD40 family MtrunA17Chr5g0436081	203	V	I	-0.628	-0.600	-0.463
M. truncatula 1739	MtrunA17Chr6g0487261	168	P	L	-0.844	1.810	-0.142
M. truncatula 1739	MtrunA17Chr7g0249241	524	T	I	-0.382	1.930	0.304
M. truncatula 1739	MtrunA17Chr7g0269361	157	E	G	-0.148	2.580	0.588
M. truncatula 1739	Putative transcription initiation factor TFIID subunit 12 MtrunA17Chr8g0343811	209	P	A	-0.642	0.200	-0.290
M. truncatula 1739	Putative tripeptidyl peptidase II MtrunA17Chr8g0345971	563	STOP	W	---	---	---
M. truncatula 1739	MtrunA17Chr8g0353481	83	S	P	-0.216	-0.850	-0.222
M. truncatula 1739	Pentatricopeptide repeat-containing protein MtrunA17Chr8g0393341	628	F	S	-1.970	-1.560	-1.984
M. truncatula 2345	MtrunA17Chr1g0190201	24	G	R	-0.249	-0.230	-0.039
M. truncatula 2345	MtrunA17Chr1g0199691	105	D	N	0.074	-0.140	0.282
M. truncatula 2345	Putative protein MtrunA17Chr1g0200031	178	T	A	-0.198	0.760	0.219
M. truncatula 2345	MtrunA17Chr2g0279331	1100	I	M	-0.596	0.080	-0.464
M. truncatula 2345	MtrunA17Chr5g0427271	151<br>153	T<br>V	A<br>A	-0.194<br>-0.577	1.270<br>-0.060	0.342<br>-0.282
M. truncatula 2345	Putative serine-threonine protein kinase MtrunA17Chr6g0465291	103	G	C	-1.228	-3.730	-1.798
		**Internode lenght**					
M. sativa IL/IC	Putative protein kinase RLK-Pelle MtrunA17Chr3g0128551	481	E	K	0.370	-0.400	0.629
